# Chrysomycins, Anti-Tuberculosis C-Glycoside Polyketides from *Streptomyces* sp. MS751

**DOI:** 10.3390/md22060259

**Published:** 2024-06-03

**Authors:** Jiaming Yu, Hui Guo, Jing Zhang, Jiansen Hu, Hongtao He, Caixia Chen, Na Yang, Fan Yang, Zexu Lin, Huanqin Dai, Liming Ouyang, Cuihua Liu, Xiaoguang Lei, Lixin Zhang, Guoliang Zhu, Fuhang Song

**Affiliations:** 1 State Key Laboratory of Bioreactor Engineering, East China University of Science and Technology, Shanghai 200237, China; 2 CAS Key Laboratory of Pathogenic Microbiology and Immunology, Institute of Microbiology, Chinese Academy of Sciences, Beijing 100101, China; 3 Beijing National Laboratory for Molecular Sciences, Key Laboratory of Bioorganic Chemistry and Molecular Engineering of Ministry of Education, Department of Chemical Biology, College of Chemistry and Molecular Engineering, and Peking-Tsinghua Center for Life Sciences, Peking University, Beijing 100871, China; 4 University of Chinese Academy of Sciences, Beijing 100049, China; 5 Technology Transfer Center, Institute of Microbiology, Chinese Academy of Sciences, Beijing 100101, China; 6 School of Medicine, University of Pittsburgh, Pittsburgh, PA 15213, USA; 7 Key Laboratory of Geriatric Nutrition and Health, Ministry of Education of China; School of Light Industry, Beijing Technology and Business University, Beijing 100048, China

**Keywords:** tuberculosis, *Streptomyces*, antibiotics, chrysomycin, biomimetic synthesis

## Abstract

A new dimeric C-glycoside polyketide chrysomycin F (**1**), along with four new monomeric compounds, chrysomycins G (**2**), H (**3**), I (**4**), J (**5**), as well as three known analogues, chrysomycins A (**6**), B (**7**), and C (**8**), were isolated and characterised from a strain of *Streptomyces* sp. obtained from a sediment sample collected from the South China Sea. Their structures were determined by detailed spectroscopic analysis. Chrysomycin F contains two diastereomers, whose structures were further elucidated by a biomimetic [2 + 2] photodimerisation of chrysomycin A. Chrysomycins B and C showed potent anti-tuberculosis activity against both wild-type *Mycobacterium tuberculosis* and a number of clinically isolated MDR *M. tuberculosis* strains.

## 1. Introduction

Tuberculosis (TB) has overtaken HIV to be ranked as the primary cause of mortality in infectious diseases globally. In 2022, the WHO reported 7.5 million TB cases, including an estimated 410,000 new cases of multidrug-resistant or rifampicin-resistant TB (MDR/RR-TB) [1], which emphasises the urgent need for novel drugs and therapies to control and ultimately clear this increasing public health threat, especially in developing countries. During the last forty years, only two new drugs, bedaquiline and delamanid, were approved for the treatment of MDR-TB and extensive drug-resistant TB (XDR-TB) in 2012 and 2014, respectively [2]. However, *M. tuberculosis* has developed an acquired resistance to these drugs very quickly [3].

Natural sources exhibit a wide range of chemical diversity and serve as an exclusive reservoir for drug discovery, with approximately 50% of clinical drugs derived from natural products or their synthetic analogue [4]. Microbial natural products have played an important role in delivering streptomycin, rifamycins, capreomycin, kanamycin, and dycloserine for the treatment of tuberculosis throughout human history. In our previous work, new antitubercular bioactive compounds, such as abyssomicins, brevianamides, lobophorins, and pluramycins, were characterised by our microbial natural library [5,6,7,8]. More specifically, a crude extract from a *Streptomyces* strain MS751 (obtained from a sediment sample retrieved at a depth of −3000 m in the South China Sea) exhibited potent antitubercular activity by using Bacillus Calmette-Guérin (BCG Pasteur 1173P2, an attenuated *Mycobacterium bovis* strain, with GFP expression vector pUV3583c-GFP) as an indicator.

## 2. Results

### 2.1. Identification, Fermentation of Strain MS751, and Purification of New Chrysomycins

**Figure 1:**
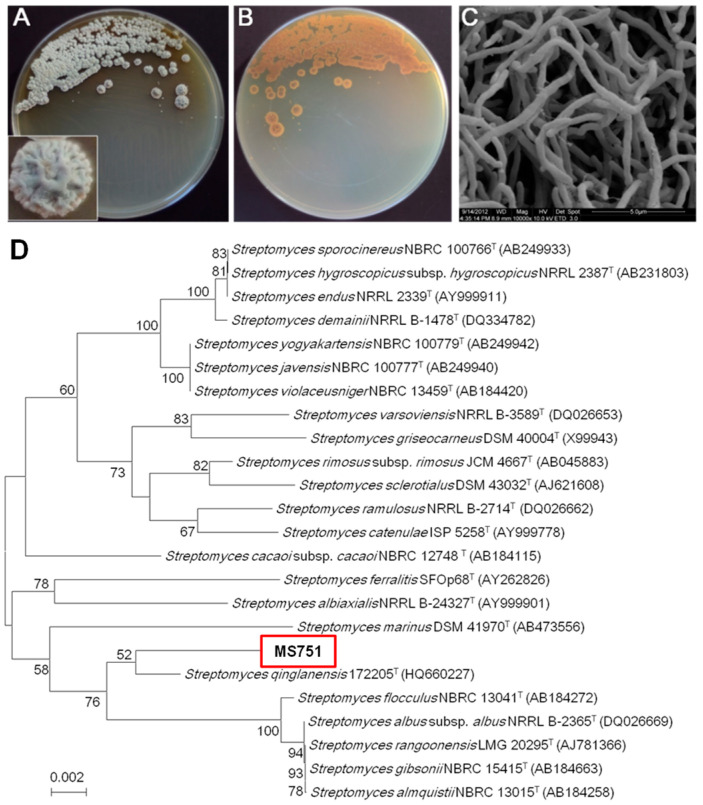
Characterisation of strain MS751. Colony front side (**A**), back side (**B**) of the strain MS751 plate, and (**C**) its scanning electron micrograph (Bar, 5 μm). (**D**) Neighbour-joining phylogenetic tree of strain MS751 based on 16S rRNA gene sequence generated by Mega4.0. Numbers at nodes indicate levels of bootstrap support (percent) based on a neighbour-joining analysis of 1000 resampled datasets; only values >50% are given. NCBI accession numbers are given in parentheses. Bar, 0.01 nucleotide substitutions per site. Also, Kutzneria albida was chosen as the outgroup.

**Figure 2:**
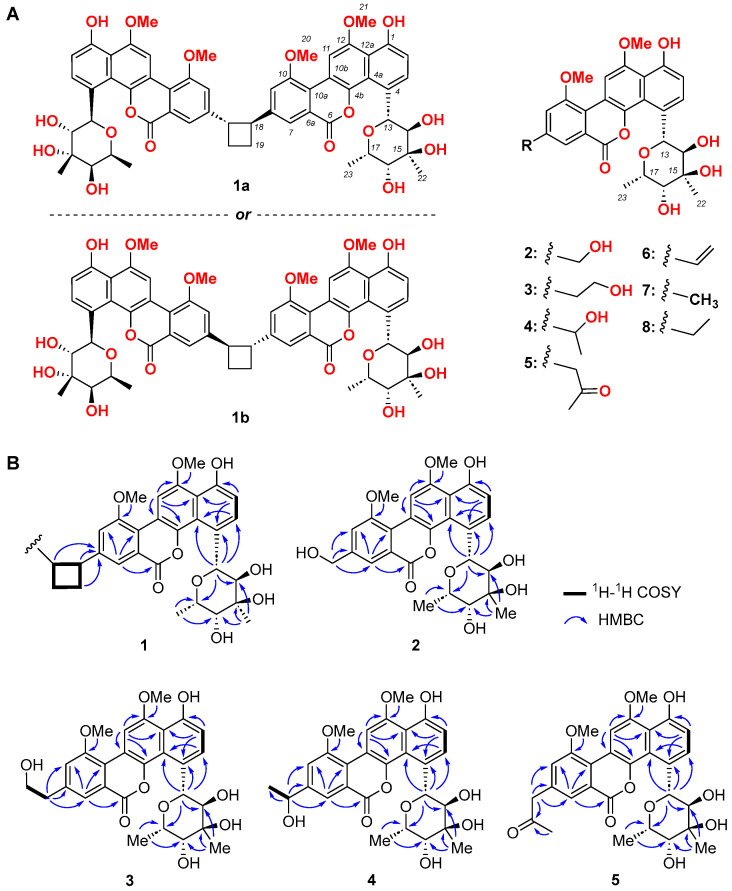
Compounds **1**–**8** isolated from *Streptomyces* sp. MS751. (**A**) structures of **1**–**8**. (**B**) 2D NMR correlations for new chrysomycins **1**–**5**.

The strain MS751 was identified as *Streptomyces* sp. By an analysis of the 16S rRNA gene sequence (Figure 1D, GenBank Accession Number: KY688100) and morphology (Figure 1A–C). A large-scale culture was performed with 30 × 1 L flasks, each charged with a 300 mL AM2 medium. After incubating for 10 d, the fermentation broth was combined and centrifuged (8000 rpm, 10 min) to yield the supernatant and mycelia. The supernatant was extracted with an equal volume of ethyl acetate (×3), and the mycelia was extracted with 1 L acetone (×3). All the organic extracts were dried in a vacuum and combined to yield the residue. The residue was subjected to reversed-phase C18 vacuum chromatography, followed by reversed-phase HPLC, to yield chrysomycins A–C and F–J (**1**–**8**) (Figure 2A). Chrysomycin F (**1**) was characterised as a new dimer of the chrysomycin class of type II polyketide by detailed spectroscopic analysis. Chrysomycins G–J (**2**–**5**) were identified as new monomeric chrysomycins, and **6**–**8** were identified as chrysomycins A–C, which were previously identified from *Streptomyces sporoverrucosus* [9]. In fact, chrysomycins A and B were first isolated from *Streptomyces* A-419 in 1955 [10].

### 2.2. Structure Elucidation of New Chrysomycins

HRESI(+)MS measurement of **1** detected a pseudo-molecular ion at *m*/*z* 1017.3531 ([M+H]^+^), which indicated a molecular formula of C_56_H_56_O_18_, requiring 29 double bond equivalents (DBE) (Figure S1a). Detailed analysis of the 1D and 2D NMR (DMSO-*d*_6_) data for **1** (Table 1) confirmed a dimeric structure, and each of the dimeric elements incorporated a 1,2,3,4-tetrasubstituted benzene, a 1,2,3,5-tetrasubstituted benzene, an isolated aromatic proton, an isolated CH_2_CH moiety, two methoxy groups, and a dimethyl-substituted pyranose moiety (Figure 2A), which could be assembled to form a chrysomycin derivative. The side chain at C8 was the moiety of CH_2_CH, based on 2D HMBC NMR data (Figure S1b–f, Table 1). The HMBC correlations from H-18/18’ to C-7/7’, C-8/8’ and C-9/9’, from H_2_-19/19’ to C-8/8’, from H-18 to C-18’, and from H-18’ to C-18 confirmed the dimeric structure of **1** (Figure 2B). The ROESY signals from H-18/18’ to H_2_-19/19’ revealed the *trans* conformation for the four-membered ring moiety (Figure S1g,h). The H_2_-19/19’ should show an identical signal in the ^1^H-NMR spectrum, if it is a *trans-*1,3-substitued dimer. Interestingly, two different signals were observed (2.33 and 2.45 ppm), which indicated that the dimer is a *trans-*1,2-substitued metabolite. All of the above-mentioned spectrum data supported the assignment of the dimeric structure for **1,** as shown in Figure 2A. However, two possible types of *trans* configuration (**1a** and **1b**) remain to be further defined.

An HRESI(+)MS measurement of **2** detected a pseudo-molecular ion at *m*/*z* 513.1758 ([M+H]^+^), which indicated a molecular formula of C_27_H_28_O_10_ (Figure S2a) and implied 14 degrees of unsaturation, suggestive of an oxidised analogue of the monomer of chrysomycin B [9] (Figure S2b–f, Table 2). The 1D NMR data of the hydroxymethyl group [*δ*_C_/*δ*_H_ 62.2/4.70 (d, *J* = 5.4 Hz)] and [*δ*_H_ 5.54 (t, *J* = 5.4 Hz)], together with the characteristic HMBC correlations from H_2_-18 to C-7, C-8, and C-9, supported the hydroxyl group at C-18 (Figure 2B), which allowed the structure to be assigned as shown (Figure 2A).

The molecular formula of **3** was revealed to be C_28_H_30_O_10_ (527.1916 [M + H]^+^, calcd. for 527.1912) through HRESI(+)MS (Figure S3a), which is suggestive of an oxidised analogue of the monomer of chrysomycin C [9] (Figure S3b–f, Table 2). The ^1^H and ^13^C NMR data of C-18−C-19−OH side chain, [*δ*_C_/*δ*_H_ 38.7/2.93 (t, *J* = 6.6 Hz), 18-CH_2_], [*δ*_C_/*δ*_H_ 61.5/2.93 (td, *J* = 6.6, 5.4 Hz), 19-CH_2_], and [*δ*_H_ 4.74 (t, *J* = 5.4 Hz), 19-OH], together with characteristic HMBC correlations from H_2_-18 to C-7, C-8, and C-9, and from H2-19 to C-8 (Figure 2B), supported the assignment of structure to be shown (Figure 2A).

The molecular formula of compound **4** was deduced as C_28_H_30_O_10_, based on HRESIMS (Figure S4a) (527.1919 ([M+H]^+^, calcd. for 527.1912) with 14 degrees of unsaturation, which is suggestive of a same molecular formula as that of **3** (Figure S4b–f, Table 2). The 1D NMR data of C-18(-OH)−C-19 side chain, [*δ*_C_/*δ*_H_ 67.7/4.92 (qd, *J* = 6.6, 5.4 Hz), 18-CH], [*δ*_C_/*δ*_H_ 25.7/1.43 (d, *J* = 6.6 Hz), 19-CH_3_], and [*δ*_H_ 4.74 (t, *J* = 5.4 Hz), 18-OH], together with characteristic HMBC correlations from H-18 to C-7, C-8, and C-9, and from H_3_-19 to C-8 (Figure 2B), supported the assignment of the structure to be shown (Figure 2A). The stereochemistry of C-18 was not determined.

An HRESI(+)MS measurement of **5** detected a pseudo-molecular ion at *m/z* 539.1913 ([M+H]^+^), which indicated a molecular formula of C_29_H_30_O_10_ (Figure S5a), requiring 15 degrees of unsaturation. A comparison of the NMR data for **5** with **2** (Figure S5b–f, Table 2), as to delivering a carbonyl-containing side chain at C-8, contained the resonances of 18-CH_2_ [*δ*_C_/*δ*_H_ 49.0/4.04, s], 19-carbonyl [*δ*_C_ 205.3] and 20-CH_3_ [*δ*_C_/*δ*_H_ 29.8/2.23, s]. With characteristic HMBC correlations from H_2_-18 to C-7, C-8, C-9, and C-19, and from H_3_-20 to C-18 and C-19 (Figure 2B), the assignment of structure to be shown was supported (Figure 2A).

### 2.3. Biomimetic [2 + 2] Photodimerisation of Chrysomycin A

**Figure 3:**
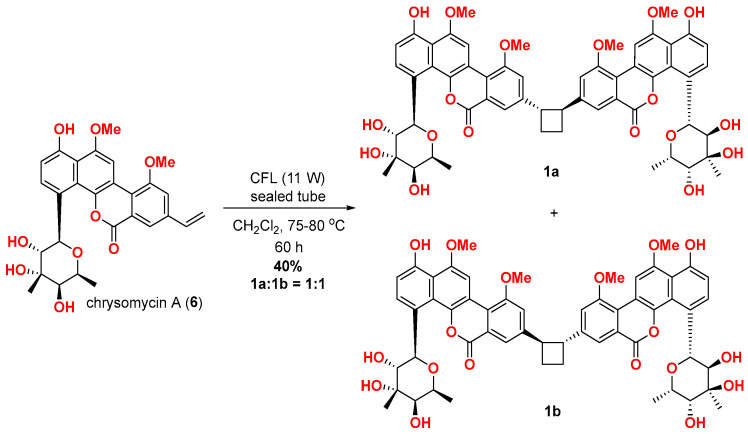
The [2 + 2] photodimerisation of chrysomycin A.

Chrysomycin F (**1**) is the bis-chrysomycin derivative possessing a cyclobutane moiety formed from chrysomycin A. To further figure out whether the stereochemistry of C-18/18’ on chrysomycin F was as indicated in **1a** or **1b**, chrysomycin A was subjected to various direct [2 + 2] photocycloaddition conditions (Table S1) [11,12,13,14,15]. As shown in Figure 3, [2 + 2] photodimerisation of chrysomycin A could be realised under visible light at 75–80 °C with CH_2_Cl_2_ as the solvent. After 60 h, chrysomycin F with the *trans-*1,2-substituted framework was obtained selectively in a 40% yield, and NMR spectra of the synthesised chrysomycin F fully matched with the ones for a natural isolate (Figure S6a,b, Table S2). At this point, two different *trans-*1,2-substitued isomers (**1a** and **1b**) should be formed in the direct photodimerisation, since the C-8 vinyl group is remote from the chiral sugar moiety. After a careful screening of UPLC separation conditions, we found both natural and synthetic chrysomycin F contained two isomers, i.e., **1a**:**1b** ratio 1:1 (Figure S7). Fortunately, these two isomers could be separated by preparative HPLC. This biomimetic [2 + 2] photodimerisation revealed that the cyclobutane ring of chrysomycin F could be directly generated from the C-8 vinyl group of chrysomycin A (Figure S8a,b, Table S3).

### 2.4. Anti-Tuberculosis Activity Evaluation

Chrysomycin A and its synthetic derivatives were reported to display significant antimicrobial activity against several *M. tuberculosis* indicators including BCG and *M. tuberculosis* H37Rv/Hr1/Hr2/Hr3/Hr4/Hr5 [16]. Thus, we also evaluate the anti-TB activities of isolated natural chrysomycins. Chrysomycins B and C displayed reduced inhibition against several *M. tuberculosis* indicators compared to chrysomycin A, with MICs ranging from 1.56 to 6.25 µg/mL (Table 3). In addition, these two compounds also exhibited antibacterial activity against gram-positive bacteria *Staphylococcus aureus* (ATCC 6538), methicillin-resistant *S. aureus* (MRSA), and *S. pneumoniae* (ATCC 49619), with MICs ranging from 3.12 to25 µg/mL but no inhibition against *C. albicans*.

## 3. Discussion

Chrysomycins belong to the benzol[*d*]naphthol [1,2*b*]pyran-6-one-C-glycoside antibiotics, which contain different side chains at the C-8 position and glycosides at the C-4 position. Fischer et al. reported the complete gene cluster for gilvocarcin V, of which there is a D-fucofuranose at the C-4 position [17]. Shepherd et al. created more gilvocarcin analogues by engineering the glycosyltransferase of GilGT [18], and Pahari synthesised the defucogilvocarcin M by in vitro enzymatic methods [19]. Previously, only chrysomycins A-C were reported with the confirmed structure, while chrysomycins D and E were only identified by LCMS/UV/DNP [9,10,20].

Chrysomycins exhibit diverse biological activities, including antibacterial [9], antitumor [21,22], anti-bacteriophage [10], and anti-neuroinflammatory activities [23]. Notably, studies have shown that chrysomycin A and its natural congeners possess significant antibacterial properties, particularly against pathogenic bacteria such as *M. tuberculosis* and *S. aureus* [24,25]. Among them, chrysomycin A demonstrated superior efficacy in treating methicillin-resistant *S. aureus* (MRSA) compared to vancomycin hydrochloride, with a MIC of 0.5 μg/mL versus 2.0 μg/mL for vancomycin hydrochloride [24]. In our earlier research, chrysomycin A exhibits potent anti-TB activity, with a MIC of 0.4 μg/mL against MDR-TB strains [16]. Structure–activity relationship studies of Chrysomycins have highlighted the crucial role of the sugar moiety in their anti-TB activity and drug resistance [16]. In this study, we investigated the antimicrobial properties of isolated natural chrysomycins. Our findings suggest that modifying the 8-vinyl group in chrysomycin A may lead to a reduction or complete loss of its antimicrobial efficacy. This observation is supported by the absence of inhibition against MTB and several other indicators when testing dimer chrysomycin F (**1**), as well as the 8-vinyl oxidised products chrysomycins H, I (**3**, **4**). Chrysomycins B (**7**) and C (**8**) displayed reduced anti-TB activity compared to chrysomycin A, with MICs ranging from 1.56–6.25 µg/mL, due to the 8-vinyl group being reduced to ethyl or replaced with methyl [16].

Cyclobutane-containing natural products have been isolated from a wide range of species, including bacteria, fungi, plants, and marine invertebrates [26,27]. Many of these natural products are thought to be generated by photochemical reactions [28]. Recently, a cyclobutane-containing gilvocarcin-type aryl-C-glycoside dimer digilvocarcin A was discovered from the soil-derived *Streptomyces* sp. OUCMDZ-945. The structure closely resembles that of the chrysomycin F obtained in this study, with the exception of the varying C-glycosides [29]. The unique 1,2-diaryl substituted cyclobutane core of chrysomycin F and digilvocarcin A rarely existed in known cyclobutane-containing natural products [26,27,30,31]. Since chrysomycin F (**1**) displayed as a pair of *trans-*1,2-substitued isomers, and that it could be formed from chrysomycin A (**6**) through a light-induced [2 + 2] cycloaddition just like digilvocarcin A [29], it is more likely a non-enzymatic product generated during the isolation process. Investigations into the chrysomycin biosynthetic gene cluster of *Streptomyces* sp. MS-751 may gain further information about this question.

## 4. Materials and Methods

### 4.1. General Experimental Section

^1^H NMR spectra were acquired using a Brucker 400 MHz, 600 MHz, or 700 MHz spectrometer (Billerica, MA, USA) with DMSO-*d*_6_ as the solvent unless otherwise stated. ^13^C NMR spectra were acquired using a Brucker 176 MHz or 201 MHz spectrometer (with complete proton decoupling). Electrospray ionisation mass spectra (ESI-MS) were recorded on an Agilent 1100 Series LC-MS (Santa Clara, CA, USA), in both positive and negative ion modes, and UPLC-MS on a Waters (Milford, MA, USA) UPLC-MS system equipped with a Waters BEH C18 1.7 μm column (2.1 × 50 mm), eluted with HPLC-grade water (solvent A) and HPLC-grade CH_3_CN (solvent B) with a flow rate of 0.3 mL/min at room temperature. HPLC was performed using an Agilent 1200 Series (Santa Clara, CA, USA) separations module equipped with a DAD detector fraction collector, controlled using ChemStation. High-resolution mass spectra were obtained using an Agilent 1200 HPLC/6520QTOFMS high-resolution mass spectrometer (Santa Clara, CA, USA) or Bruker APEX Flash chromatography (Billerica, MA, USA). Optical rotations were recorded on an Insmark^®^ digital polarimeter (Shanghai, China) at 589 nm and are recorded as [*α*${]}_{D}^{25}$.

### 4.2. Characterisation of Streptomyces sp. Strain MS751

Strain MS751 (*Streptomyces* sp.) was obtained from a sediment sample that was collected from the South China Sea and was characterised as a *Streptomyces* sp. using 16S rRNA gene sequence analysis (GenBank accession no. KY688100). The strain has been submitted to the China General Microbiological Culture Collection Centre (accession no. 6299), which is a member of the World Data Centre for Microorganisms (WDCM 550).

### 4.3. Cultivation, Extraction, and Compounds Purification

Strain MS751 was cultivated on a Gauze–asparagine (GA) agar plate at 28 °C (Table S4); 10 × 250 mL Erlenmeyer flasks, each containing 40 mL of ISP2 liquid medium, were inoculated with MS751 and incubated at 28 °C (220 rpm) for 3 days. Aliquots (5 mL) of the seed were used to inoculate 30 × 1 L Erlenmeyer flasks, each containing 300 mL of AM2 liquid medium, and the flasks were incubated at 28 °C (140 rpm) for 10 days. The culture broths were pooled and then subjected to centrifugation to separate into a supernatant and mycelial fraction. The mycelial was extracted with acetone (3 × 600 mL) and further reduced to dryness in vacuo to yield the crude extract F1 (2.2 g). The supernatant was extracted with an equal volume of ethyl acetate (×3) and concentrated to yield the crude extract F2 (0.8 g). The two extracts were combined and subjected to a reduced pressure reversed phase C18 silica gel column using a gradient of MeOH in H_2_O (10%, 30%, 40%, 60%, 80%, 90%, and 100%) to afford 7 subfractions. Subfraction RP7 was further purified by HPLC (Zorbax C18 5 μm 250 × 9.4 mm column, 2.5 mL/min, 70% MeCN in H_2_O) to yield compound **1** (1.7 mg). Subfraction RP4 was subsequently subjected to HPLC fractionation (Agilent Zorbax SB-C18 5 μm 250 × 9.4 mm column, 2.0 mL/min, 65% MeOH in H_2_O) to yield compound **2** (2.8 mg). Subfraction RP3 was purified by HPLC (Agilent Zorbax SB-C18 5 μm 250 × 9.4 mm column, 2.0 mL/min, 60% MeOH in H_2_O) to yield compounds **3** (4.7 mg), **4** (2.5 mg), and **5** (6.4 mg). The second fraction was subsequently subjected to HPLC fractionation (Agilent Zorbax SB-C18 5 μm 250 × 9.4 mm column, 2.0 mL/min, 65% MeOH in H_2_O) to yield compounds **6** (20.3 mg), **7** (14.5 mg), and **8** (8.4 mg).

Chrysomycin F (**1**) (*trans*-dimers **1a**:**1b** = 1:1): light yellow powder; NMR (600 MHz, DMSO-*d*_6_) see Table 1; HRESI(+)MS *m*/*z* 1017.3531 (calc for C_56_H_57_O_18_, 1017.3539)

Chrysomycin G (**2**): light yellow powder; [*α*${]}_{D}^{25}$ −15.0 (MeOH; *c* 0.06); NMR (600 MHz, DMSO-*d*_6_) see Table 2; HRESI(+)MS *m*/*z* 513.1758 (calc for C_27_H_29_O_10_, 513.1755)

Chrysomycin H (**3**): light yellow powder; [*α*${]}_{D}^{25}$ −18.5 (MeOH; *c* 0.025); NMR (600 MHz, DMSO-*d*_6_) see Table 2; HRESI(+)MS *m*/*z* 527.1916 (calc for C_28_H_31_O_10_, 527.1912)

Chrysomycin I (**4**): light yellow powder; [*α*${]}_{D}^{25}$ −22.5 (MeOH; *c* 0.025); MR (600 MHz, DMSO-*d*_6_) see Table 2; HRESI(+)MS *m*/*z* 527.1919 (calc for C_28_H_31_O_10_, 527.1912)

Chrysomycin J (**5**): light yellow powder; [*α*${]}_{D}^{25}$ −32.0 (MeOH; *c* 0.025); NMR (600 MHz, DMSO-*d*_6_) see Table 2; HRESI(+)MS *m*/*z* 539.1913 (calc for C_29_H_31_O_10_, 539.1912)

### 4.4. Synthesis, Isolation, and Characterisation of trans-Dimers 1a and 1b

To a sealed tube were added chrysomycin A (4.5 mg, 0.0089 mmol) and 0.5 mL of CH_2_Cl_2_ under argon. The solution obtained was agitated at a temperature range of 75–80 °C with the irradiation of an 11 W compact fluorescent lightbulb. After 60 h, the reaction was cooled and then concentrated. The residue was purified by silica gel column chromatography (CH_2_Cl_2_/MeOH = 50/1 to 10/1) to give chrysomycin F (1.8 mg, 40%, **1a**:**1b** = 1:1) as a light-yellow powder. The two *trans*-dimers, **1a** and **1b,** were further separated by preparative HPLC (XBridge C18 5 μm, 19 × 150 mm column, 20 mL/min, 0–19 min 38% CH_3_CN in H_2_O, then 19–30 min 40% CH_3_CN in H_2_O), to yield **1a** (0.73 mg) and **1b** (0.75 mg).

Synthetic chrysomycin F (**1a**:**1b** = 1:1): light yellow powder; ^1^H NMR (400 MHz, DMSO-*d*_6_) and ^13^C NMR (201 MHz, DMSO-*d*_6_) see Table S2; HRESI(+)MS *m*/*z* 1039.3352 (calc for C_56_H_56_NaO_18_, 1039.3359).

Synthetic *trans*-dimer **1a** (or **1b**): light yellow powder; [*α*${]}_{D}^{25}$ +112.2 (*c* 0.03, CHCl_3_); NMR (700 MHz, DMSO-*d*_6_) see Table S3; HRESI(+)MS *m*/*z* 1039.3359 (calc for C_56_H_56_NaO_18_, 1039.3359).

Synthetic *trans*-dimer **1b** (or **1a**): light yellow powder; [*α*${]}_{D}^{25}$ −150.7 (*c* 0.03, CHCl_3_); NMR (700 MHz, DMSO-*d*_6_) see Table S3; HRESI(+)MS *m*/*z* 1039.3357 (calc for C_56_H_56_NaO_18_, 1039.3359).

### 4.5. Bioassays for Purified Compounds

The microbe strains used for anti-tuberculosis assays are *M. bovis* BCG Pasteur 1173P2, *M. tuberculosis* H37Rv, *M. Tuberculosis* Hr1, *M. Tuberculosis* Hr2, *M. Tuberculosis* Hr3, *M. Tuberculosis* Hr4, *M. Tuberculosis* Hr5, and *Mycobacterium smegmatis* mc215*5*, respectively. The Bacille *Calmette–Guerin* (Pasteur 1173P2) assay was carried out utilising a procedure in our previous report [16]. The assay for anti-*M. tuberculosis* (H37Rv/Hr1/Hr2/Hr3/Hr4/Hr5) was carried out by a modified M24-A2 method from the Clinical and Laboratory Standards Institute [32].

The assay for anti-*M*. *smegmatis* was performed according to the Clinical and Laboratory Standards Institute, and some modifications were made. *M. smegmatis* mc2155 was precultured at 37 °C in Middlebrook 7H9 broth (Difco) for 24 h. Then, it was diluted with 7H9 broth to yield a broth of CFU of 10^6^. The compounds were prepared at 100× stocks in DMSO. Serial dilutions of the compounds were prepared in the same solvent and added to the wells in a 2 mL volume. Isoniazid (purchased from Amresco (Solon, OH, USA), ultrapure grade) was used as a positive control for this assay. The plates were placed in a 37 °C incubator for 16 h. Subsequently, 10 μL of AlamarBlue was added and the plates were further incubated for 2 h. The inhibitory effect was determined by measuring fluorescence using an envision 2103 multilabel reader (Perkin-Elmer Life Sciences, Shelton, CT, USA) with excitation at 530 nm and emission at 590 nm. MIC here is defined as the minimum concentration of compound that inhibits more than 90% of bacterial growth reflected by ODs.

Assays for anti*-Staphylococcus aureus,* methicillin-resistant *Staphylococcus aureus,* and *Staphylococcus pneumoniae* ATCC 49619 screening assays were performed according to the CLSI Antimicrobial Susceptibility Testing Standards and our previous report [6]. Antifungal bioassays were performed according to a modified protocol of the CLSI M-27A [33] methods using the fungus *Candida albicans* (SC 5314).

## 5. Conclusions

In conclusion, we have isolated and fully characterised a number of new C-glycoside polyketide chrysomycin natural products using the bioassay-guided separation from *Streptomyces sp.* MS751. Chrysomycin F (**1**) shows a unique dimeric structure containing a *trans-*1,2-disubstitued cyclobutane motif, which was further confirmed by extensive spectroscopic analysis, as well as a biomimetic transformation from the monomeric precursor through [2 + 2] photodimerisation. Chrysomycins B and C show potent anti-TB activities against a panel of clinically isolated MDR TB strains. Our efforts to develop novel anti-TB drug candidates sourced from chrysomycins are presently underway and will be disclosed in due course.

## Figures and Tables

**Table 1:** ^1^H, ^13^C NMR and HMBC data for chrysomycin F (**1**) in DMSO-*d*_6._

Position	δ_H_,^a^ *mult* (*J* in Hz)	δ_C_^b^	HMBC (^1^H→^13^C)
1/1’		153.2, C	
2/2’	6.96, d (8.4)	112.0, CH	1/1’, 4/4’, 12a/12a’
3/3’	7.83, d (8.4)	129.3, CH	1/1’, 4a/4a’, 13/13’
4/4’		128.1, C	
4a/4a’		125.2, C	
4b/4b’		142.3, C	
6/6’		159.9, C	
6a/6a’		121.9, C	
7/7’	7.88/7.89, d (1.2)	119.0/118.9, CH	6/6’, 9/9’, 10a/10a’, 18/18’
8/8’		146.2, C	
9/9’	7.58/7.61, d (1.2)	116.5, CH	7/7’, 10/10’, 10a/10a’, 18/18’
10/10’		157.4/157.3, C	
10a/10a’		121.8, C	
10b/10b’		113.3, C	
11/11’	8.49, s	101.6, CH	4b/4b’, 10a/10a’, 10b/10b’, 12/12’, 12a/12a
12/12’		151.9, C	
12a/12a’		115.1, C	
13/13’	6.01, d (9.6)	74.6, CH	3/3’, 4/4’, 4a/4a’, 14/14’, 17/17’
14/14’	3.66, dd (9.6, 8.4)	72.6, CH	13/13’
15/15’		73.1, C	
16/16’	3.13, d (7.8)	75.8, CH	15/15’
17/17’	4.50, brq (6.0)	70.7, CH	13/13’, 16/16’, 23/23’
18/18’	3.96, m	47.0/46.9, CH	8/8’, 19/19’
19a/19’a	2.33, m	25.4/25.5, CH_2_	18/18’
19b/19’b	2.45, m		18/18’
20/20’	4.15/4.14, s	56.7/56.7, CH_3_	10/10’
21/21’	4.11, s	56.3, CH_3_	12/12’
22/22’	1.24, s	23.9, CH_3_	14/14’, 15/15’, 16/16’
23/23’	1.00/1.01, d (6.6)	17.1, CH_3_	16/16’, 17/17’
1/1’-OH	9.81, s		1/1’, 2/2’, 12a/12a’
14/14’-OH	4.16, overlap (8.4)		
15/15’-OH	4.18, s		14/14’, 16/16’
16/16’-OH	4.57, d (7.8)		16/16’

^a 1^H (600 MHz), ^b 13^C (150 MHz).

**Table 2:** ^1^H and ^13^C NMR data for chrysomycin G−J (**2**−**5**) in DMSO-*d*_6._

Position	Chrysomycin G (2)		Chrysomycin H (3)		Chrysomycin I (4)		Chrysomycin J (5)	
	δ_H_,^a^ *mult* (*J* in Hz)	δ_C_^b^	δ_H_,^a^ *mult* (*J* in Hz)	δ_C_^b^	δ_H_,^a^ *mult* (*J* in Hz)	δ_C_^b^	δ_H_,^a^ *mult* (*J* in Hz)	δ_C_^b^
1		153.2, C		153.2, C		153.3, C		153.2, C
2	6.97, d (8.4)	112.0, CH	6.96, d (8.4)	111.9, CH	6.96, d (8.4)	112.1, CH	6.97, d (8.4)	112.0, CH
3	7.84, d (8.4)	129.3, CH	7.83, d (8.4)	129.3, CH	7.83, d (8.4)	129.4, CH	7.84, d (8.4)	129.3, CH
4		128.0, C		128.0, C		128.1, C		128.1, C
4a		125.2, C		125.2, C		125.3, C		125.2, C
4b		142.3, C		142.2, C		142.4, C		142.3, C
6		160.0, C		160.0, C		160.2, C		159.9, C
6a		121.7, C		121.5, C		121.7, C		121.6, C
7	7.96, s	118.4, CH	7.84, s	121.4	7.95, d (1.8)	117.7, CH	7.80, d (1.2)	122.4, CH
8		145.4, C		142.7, C		150.1, C		137.6, C
9	7.60, s	115.8, CH	7.55, d (1.2)	119.1	7.61, d, (1.8)	115.4, CH	7.49, d (1.2)	119.7, CH
10		157.1, C		156.9, C		157.2, C		156.9, C
10a		121.9, C		121.4, C		122.0, C		121.9, C
10b		113.4, C		113.4, C		113.5, C		113.2, C
11	8.52, s	101.7, CH	8.50, s	101.7, CH	8.49, s	101.8, CH	8.48, s	101.6, CH
12		151.9, C		151.9, C		152.0, C		151.8, C
12a		115.1, C		115.1, C		115.2, C		115.1, C
13	6.03, d (9.6)	74.6, CH	6.03, d (9.6)	74.6, CH	6.02, d (9.6)	74.7, CH	6.03, d (9.6)	74.6, CH
14	3.69, dd (9.6, 8.4)	72.5, CH	3.67, dd (9.6, 8.4)	72.6, CH	3.68, dd (9.6, 8.4)	72.7, CH	3.67, dd (9.6, 8.4)	72.6, CH
15		73.1, C		73.1, C		73.3, C		73.1, C
16	3.15, d (7.8)	75.8, CH	3.14, d (7.8)	75.8, CH	3.15, d (7.8)	75.9, CH	3.14, d (7.8)	75.8, CH
17	4.52, q (6.6)	70.7, CH	4.51, q (6.6)	70.7, CH	4.52, q (6.6)	70.8, CH	4.51, q (6.6)	70.7, CH
18	4.70, d (5.4)	62.2, CH_2_	2.93, t (6.6)	38.7, CH_2_	4.92, qd (6.6, 4.8)	67.7, CH	4.04, s	49.0, CH_2_
19			3.75, td (6.6, 5.4)	61.5, CH_2_	1.43, d (6.6)	25.7, CH_3_		205.3, C
19’							2.23, s	
20	4.13, s	56.6, CH_3_	4.13, s	56.7, CH_3_	4.13, s	56.8, CH_3_	4.10, s	29.8, CH_3_
21	4.13, s	56.4, CH_3_	4.13, s	56.4, CH_3_	4.11, s	56.5, CH_3_	4.12, s	56.7, CH_3_
22	1.26, s	23.9, CH_3_	1.25, s	23.9, CH_3_	1.25, s	24.0, CH_3_	1.26, s	56.3, CH_3_
23	1.01, d (6.6)	17.1, CH_3_	1.01, d (6.6)	17.1, CH_3_	1.01, d (6.6)	17.2, CH_3_	1.02, d (6.6)	23.9, CH_3_
1-OH	9.83, s		9.82, s		9.82, s		9.82, s	17.1, CH_3_
14-OH	4.17, d (8.4)		4.17, d (8.4)		4.19, d (8.4)		4.18, d (8.4)	
15-OH	4.20, s		4.19, s		4.22, s		4.20, s	
16-OH	4.57, d (7.8)		4.57, d (7.8)		4.63, d (7.8)		4.58, d (7.8)	
18-OH	5.54, t (5.4)				5.55, d (4.8)			
19-OH			4.75, t (5.4)					

^a 1^H (600 MHz), ^b 13^C (150 MHz).

**Table 3:** Antimicrobial activity of compounds **1**–**5**, **7,** and **8**.

Microorganism (Strain)	Minimum Inhibitory Concentration (μg/mL)							
	1	2	3	4	5	7	8	Control
*M. bovis* BCG	50.0	12.5	>100	>100	>100	6.25	3.13	0.05 ^[a]^
*M. tuberculosis* H37Rv	>100	50.0	>100	>100	>100	1.56	1.56	0.02 ^[b]^
*M. tuberculosis* (Hr1)	>100	>100	>100	>100	>100	1.56	1.56	1.00 ^[b]^
*M. tuberculosis* (Hr2)	>100	>100	>100	>100	>100	1.56	1.56	2.00 ^[b]^
*M. tuberculosis* (Hr3)	>100	>100	>100	>100	>100	3.12	1.56	0.50 ^[b]^
*M. tuberculosis* (Hr4)	>100	>100	>100	>100	>100	1.56	3.12	1.00 ^[b]^
*M. tuberculosis* (Hr5)	>100	>100	>100	>100	>100	3.12	1.56	2.00 ^[b]^
*M. smegmatis* mc2155	100	>100	>100	>100	>100	1.56	3.13	3.13 ^[a]^
MRSA	>25.0	>100	>100	>100	>100	6.25	6.25	1.00 ^[c]^
*S. aureus* ATCC 6538	>25.0	>100	>100	>100	>100	3.13	6.25	1.00 ^[c]^
S*. pneumoniae* ATCC 49619	>100	50.0	>100	>100	>100	3.13	25.0	5.00 ^[d]^
*C. albicans*	>100	>100	>100	>100	>100	>100	>100	0.02 ^[e]^

[a] Isoniazid. [b] Rifampicin. [c] Vancomycin. [d] Chloramphenicol. [e] Ketoconazole.

## Data Availability

The data are included in the article and the Supplementary Material; further inquiries can be directed to the corresponding author.

## Supplementary Materials

The following supporting information can be downloaded at https://www.mdpi.com/article/10.3390/md22060259/s1, Supplementary Text: Purification of Chrysomycins, and The assay for Anti-*Mycobacterium smegmatis*; Figures S1a–S5f: HRESIMS, 1D and 2D NMR spectra of new compounds **1**–**5**; Figure S6: 1D NMR spectra of synthetic chrysomycin F; Figure S7: Preparative HPLC chromatogram of synthetic chrysomycin F; Figures S8 and S9: 1D NMR spectra of synthetic trans-dimer **1a** and **1b**; Table S1: Screening of conditions for the [2 + 2] photodimerisation of chrysomycin A; Table S2: 1D NMR data comparison of natural and synthetic chrysomycin F; Table S3: NMR data for synthetic trans-dimers **1a** and **1b** in DMSO-*d*_6_; Table S4: Composition of the cluture media.
