# Supplementary material for: Chrysomycins, Anti-Tuberculosis C-Glycoside Polyketides from Streptomyces sp. MS751
Source: Mar Drugs. 2024 Jun 3;22(6):259. doi: 10.3390/md22060259 (PMC11204892; doi:10.3390/md22060259)
Supplement: Supplementary file 1 [file marinedrugs-22-00259-s001.zip › marinedrugs-3019980-supplementary.pdf]

Supplemental Material

Chrysomycins, anti-tuberculosis C-glycoside polyketides from *Streptomyces* sp. MS751

Jiaming Yu [1,‡], Hui Guo [2,‡], Jing Zhang [3], Jiansen Hu [2,4], Hongtao He [2], Caixia Chen [5,6], Na Yang [2], Fan Yang [3], Zexu Lin [1], Huanqin Dai [2], Liming Ouyang [1], Cuihua Liu [2], Xiaoguang Lei [3], Lixin Zhang [1,2], Guoliang Zhu [1,*] and Fuhang Song [2,7,*]

1 State Key Laboratory of Bioreactor Engineering, East China University of Science and Technology, Shanghai 200237, China;
2 CAS Key Laboratory of Pathogenic Microbiology and Immunology, Institute of Microbiology, Chinese Acade-my of Sciences, Beijing 100101, China;
3 Beijing National Laboratory for Molecular Sciences, Key Laboratory of Bioorganic Chemistry and Molecular Engineering of Ministry of Education, Department of Chemical Biology, College of Chemistry and Molecular Engineering, and Peking-Tsinghua Center for Life Sciences, Peking University, Beijing 100871, China;
4 University of Chinese Academy of Sciences, Beijing 100049, China;
5 Technology Transfer Center, Institute of Microbiology, Chinese Academy of Sciences, Beijing 100101, China;
6 School of Medicine, University of Pittsburgh, Pennsylvania 15213, USA;
7 Key Laboratory of Geriatric Nutrition and Health, Ministry of Education of China; School of Light Industry, Beijing Technology and Business University, Beijing 100048, China.
‡ The authors contributed to this work equally.
* Correspondence: zhuguoliang@ecust.edu.cn (G.Z.); songfuhang@btbu.edu.cn (F.S.)

Table of Contents

Figure S1a. HRESIMS spectrum of **1** 3
Figure S1b. ^{1}H NMR spectrum (600 MHz, DMSO-d_6) of **1** 3
Figure S1c. ^{13}C NMR spectrum (150 MHz, DMSO-d_6) of **1** 4
Figure S1d. COSY spectrum (600 MHz, DMSO-d_6) of **1** 4
Figure S1e. ^{1}H – ^{13}C HSQC spectrum (600 MHz, DMSO-d_6) of **1** 5
Figure S1f. ^{1}H – ^{13}C HMBC spectrum (600 MHz, DMSO-d_6) of **1** 5
Figure S1g. ROESY spectrum (600 MHz, DMSO-d_6) of **1** 6
Figure S1h. Expansion of ROESY spectrum (600 MHz, DMSO-d_6) of **1** 6
Figure S2a. HRESIMS spectrum of **2** 7
Figure S2b. ^{1}H NMR spectrum (600 MHz, DMSO-d_6) of **2** 7
Figure S2c. ^{13}C NMR spectrum (150 MHz, DMSO-d_6) of **2** 8
Figure S2d. COSY spectrum (600 MHz, DMSO-d_6) of **2** 8
Figure S2e. ^{1}H – ^{13}C HSQC spectrum (600 MHz, DMSO-d_6) of **2** 9
Figure S2f. ^{1}H – ^{13}C HMBC spectrum (600 MHz, DMSO-d_6) of **2** 9
Figure S3a. HRESIMS spectrum of **3** 10
Figure S3b. ^{1}H NMR spectrum (600 MHz, DMSO-d_6) of **3** 10
Figure S3c. ^{13}C NMR spectrum (150 MHz, DMSO-d_6) of **3** 11
Figure S3d. COSY spectrum (600 MHz, DMSO-d_6) of **3** 11
Figure S3e. ^{1}H – ^{13}C HSQC spectrum (600 MHz, DMSO-d_6) of **3** 12
Figure S3f. ^{1}H – ^{13}C HMBC spectrum (600 MHz, DMSO-d_6) of **3** 12
Figure S4a. HRESIMS spectrum of **4** 13
Figure S4b. ^{1}H NMR spectrum (600 MHz, DMSO-d_6) of **4** 13
Figure S4c. ^{13}C NMR spectrum (150 MHz, DMSO-d_6) of **4** 14
Figure S4d. COSY spectrum (600 MHz, DMSO-d_6) of **4** 14
Figure S4e. ^{1}H – ^{13}C HSQC spectrum (600 MHz, DMSO-d_6) of **4** 15
Figure S4f. ^{1}H – ^{13}C HMBC spectrum (600 MHz, DMSO-d_6) of **4** 15

Figure S5a. HRESIMS spectrum of **5** 16
Figure S5b. ^{1}H NMR spectrum (600 MHz, DMSO-d_6) of **5** 16
Figure S5c. ^{13}C NMR spectrum (150 MHz, DMSO-d_6) of **5** 17
Figure S5d. COSY spectrum (600 MHz, DMSO-d_6) of **5** 17
Figure S5e. ^{1}H – ^{13}C HSQC spectrum (600 MHz, DMSO-d_6) of **5** 18
Figure S5f. ^{1}H – ^{13}C HMBC spectrum (600 MHz, DMSO-d_6) of **5** 18
Table S1. Screening of conditions for the [2+2] photodimerization of chrysomycin A 18
Figure S6a. ^{1}H NMR spectrum (400 MHz, DMSO-d_6) of synthetic chrysomycin F (**1**) 19
Figure S6b. ^{13}C NMR spectrum (201 MHz, DMSO-d_6) of synthetic chrysomycin F (**1**) 20
Table S2. ^{1}H and ^{13}C NMR data comparison of natural and synthetic chrysomycin F (**1**) in DMSO-d_6 20
Figure S7. UPLC chromatogram of synthetic chrysomycin F (**1**) 22
Figure S8a. ^{1}H NMR spectrum (700 MHz, DMSO-d6) of synthetic *trans*-dimer **1a** (or **1b**) 22
Figure S8b. ^{13}C NMR spectrum (176 MHz, DMSO-d_6) of synthetic *trans*-dimer **1a** (or **1b**) 23
Figure S9a. ^{1}H NMR spectrum (700 MHz, DMSO-d_6) of synthetic *trans*-dimer **1b** (or **1a**) 23
Figure S9b. ^{13}C NMR spectrum (176 MHz, DMSO-d_6) of synthetic *trans*-dimer **1b** (or **1a**) 24
Table S3. NMR data for synthetic *trans*-dimers **1a** and **1b** in DMSO-d_6 25
Table S4. Composition of the cluture media 26

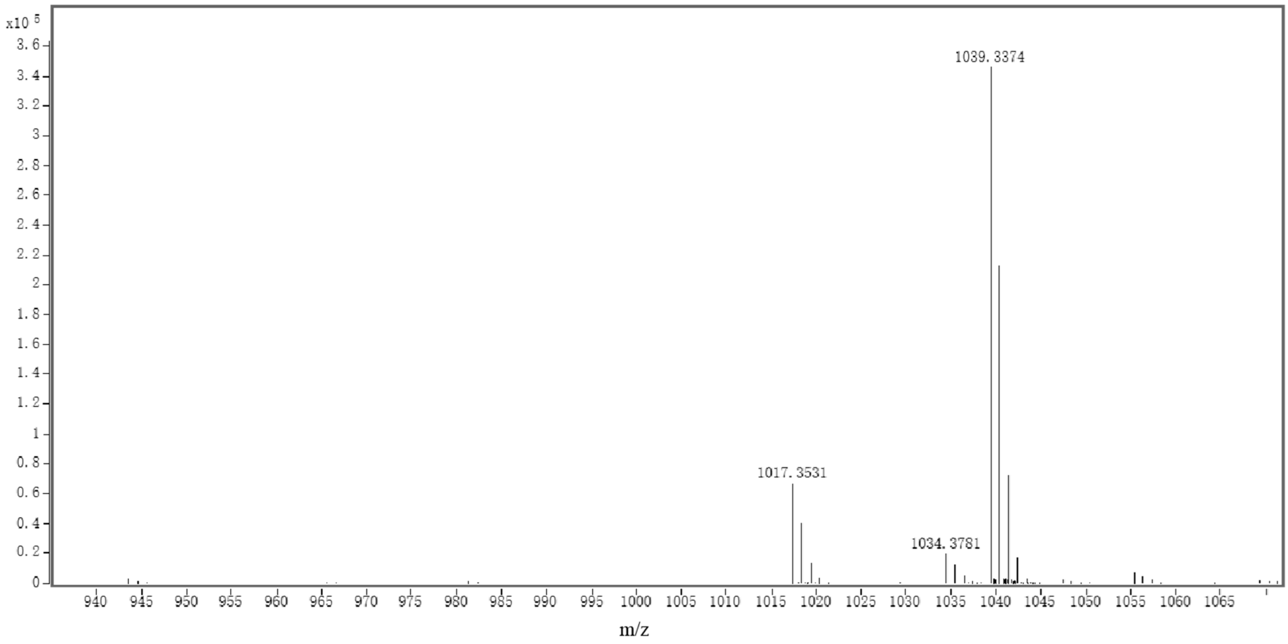

Figure S1a. HRESIMS spectrum of **1**

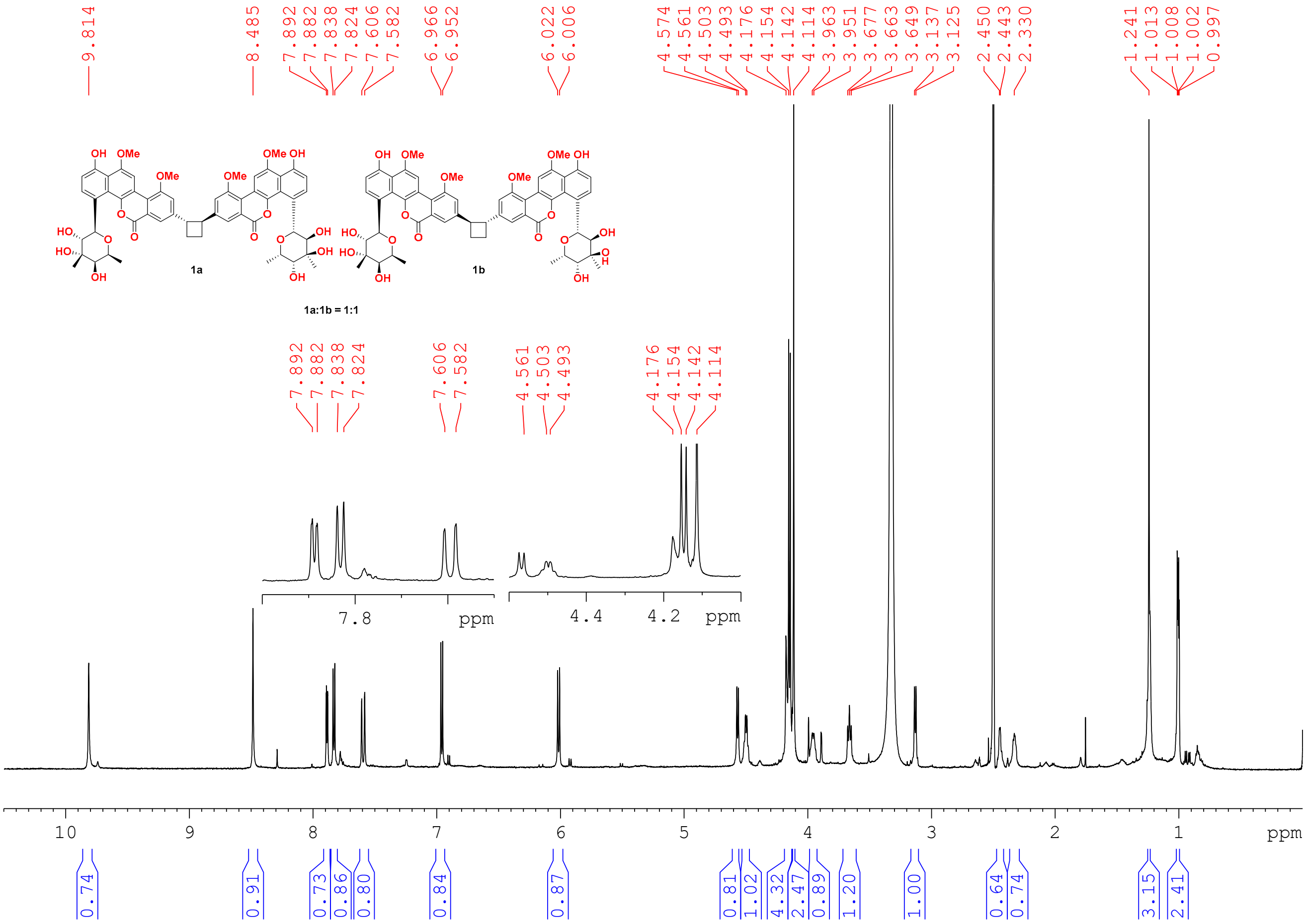

Figure S1b. ^1H NMR spectrum (600 MHz, DMSO-d_6) of **1**

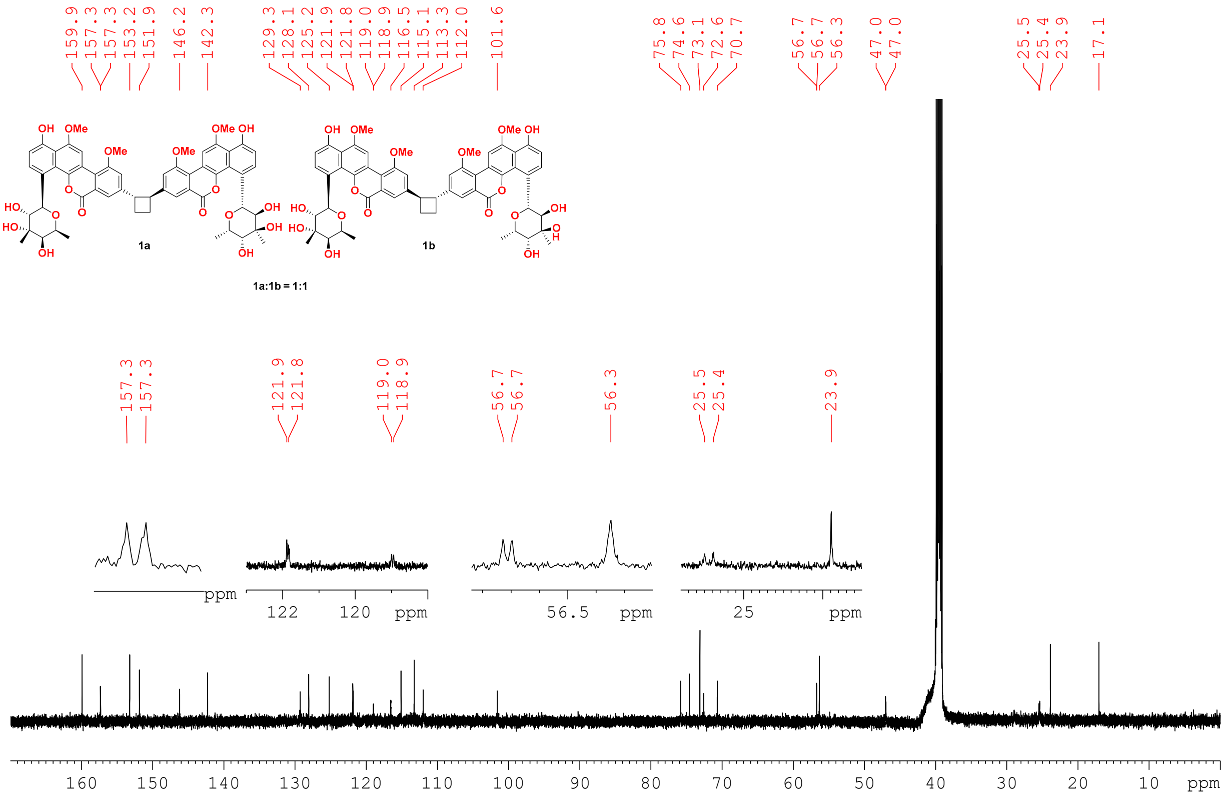

Figure S1c. ^{13}C NMR spectrum (150 MHz, DMSO-d_6) of **1**

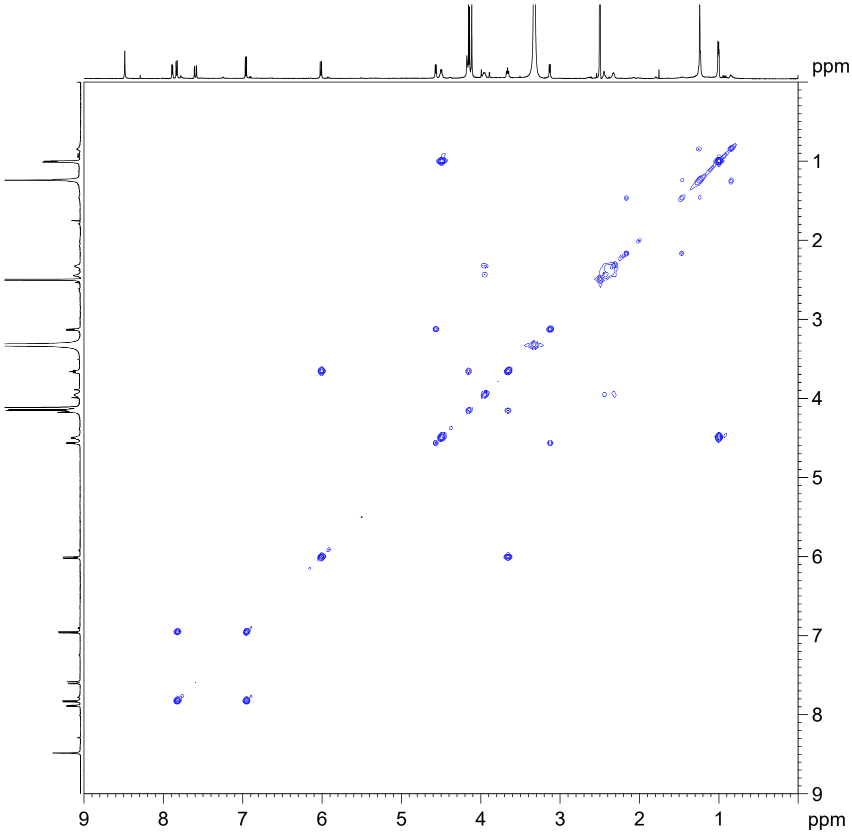

Figure S1d. COSY spectrum (600 MHz, DMSO-d_6) of **1**

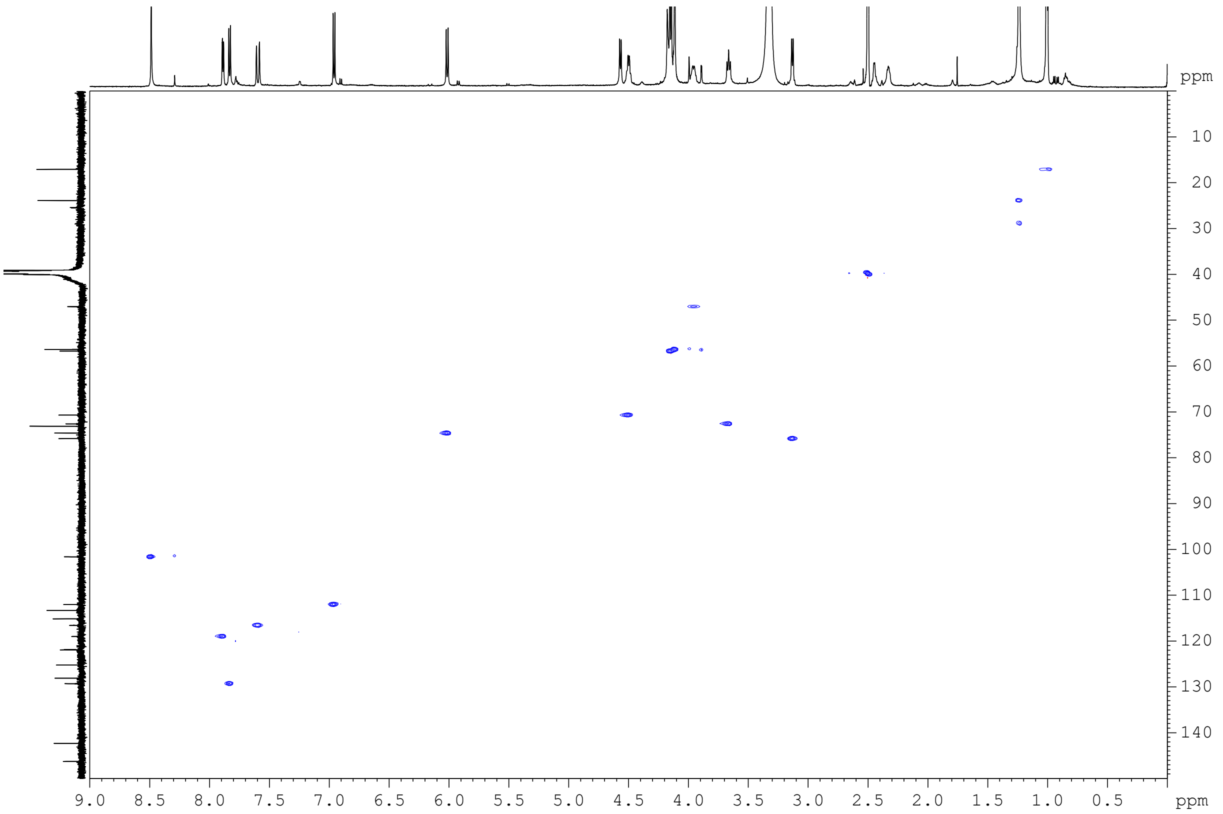

Figure S1e. $^{1}H – {}^{13}C$ HSQC spectrum (600 MHz, DMSO-*d*$_6$) of **1**

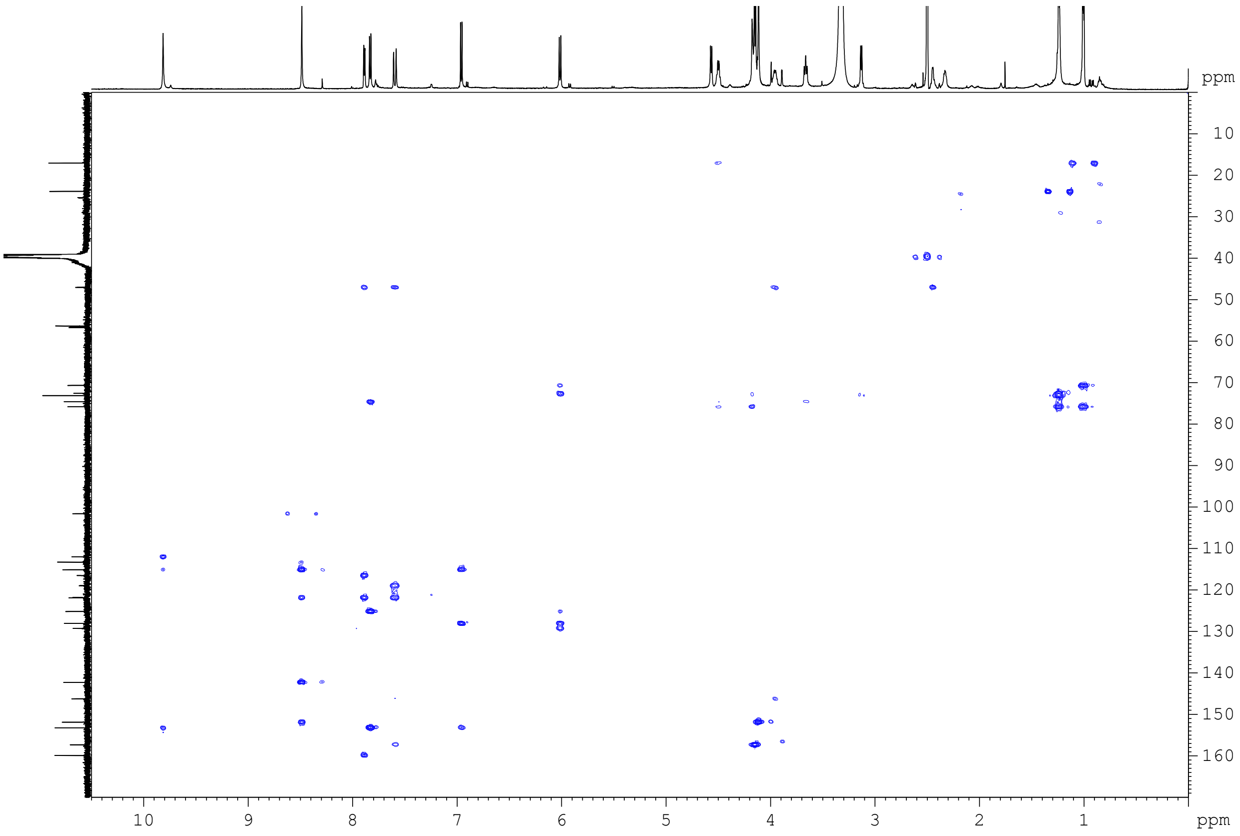

Figure S1f. $^{1}H – {}^{13}C$ HMBC spectrum (600 MHz, DMSO-*d*$_6$) of **1**

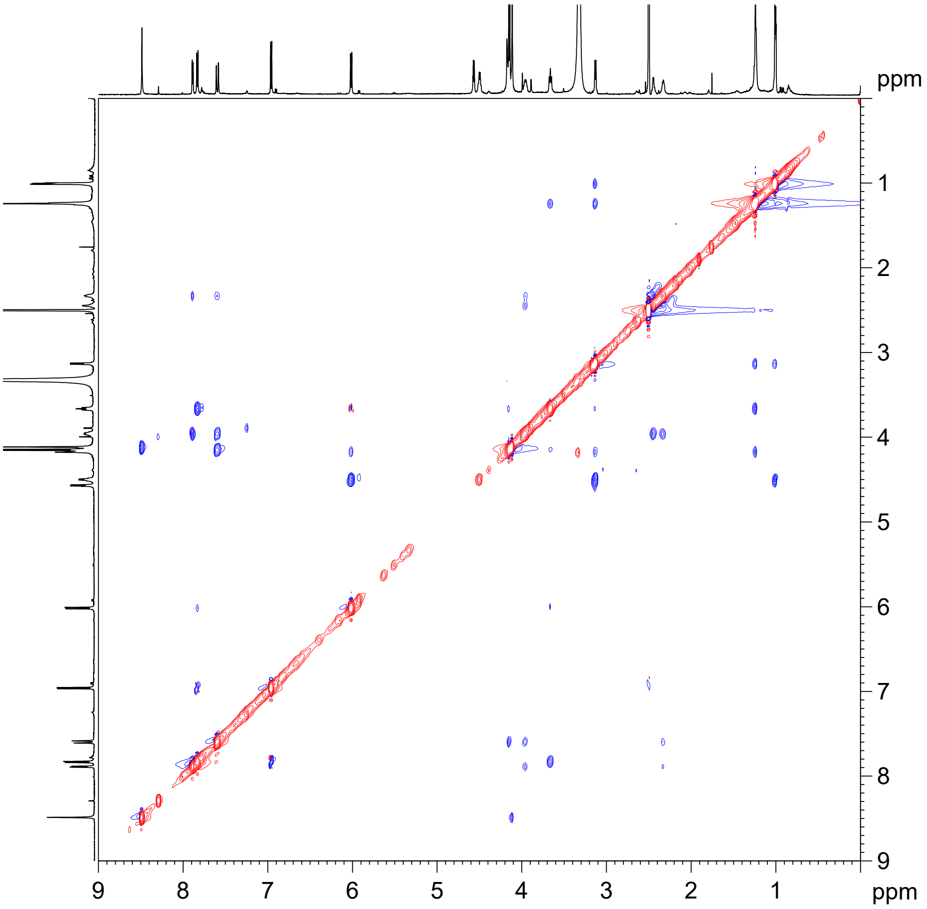

Figure S1g. ROESY spectrum (600 MHz, DMSO-d_6) of **1**

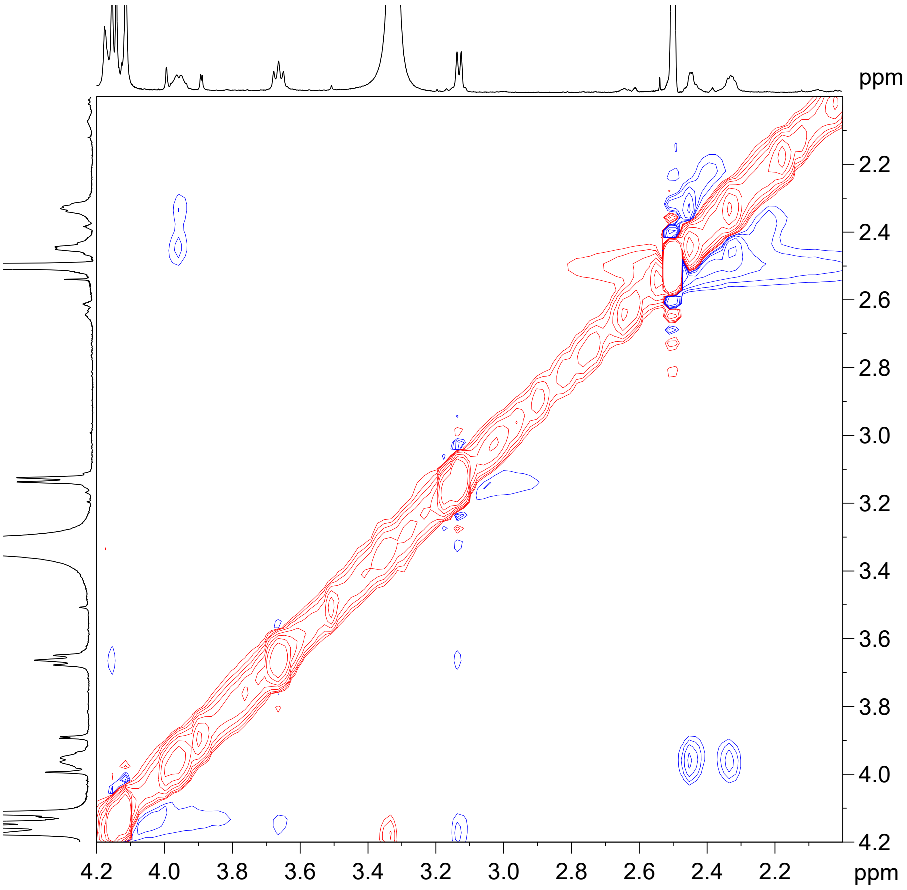

Figure S1h. Expansion of ROESY spectrum (600 MHz, DMSO-d_6) of **1**

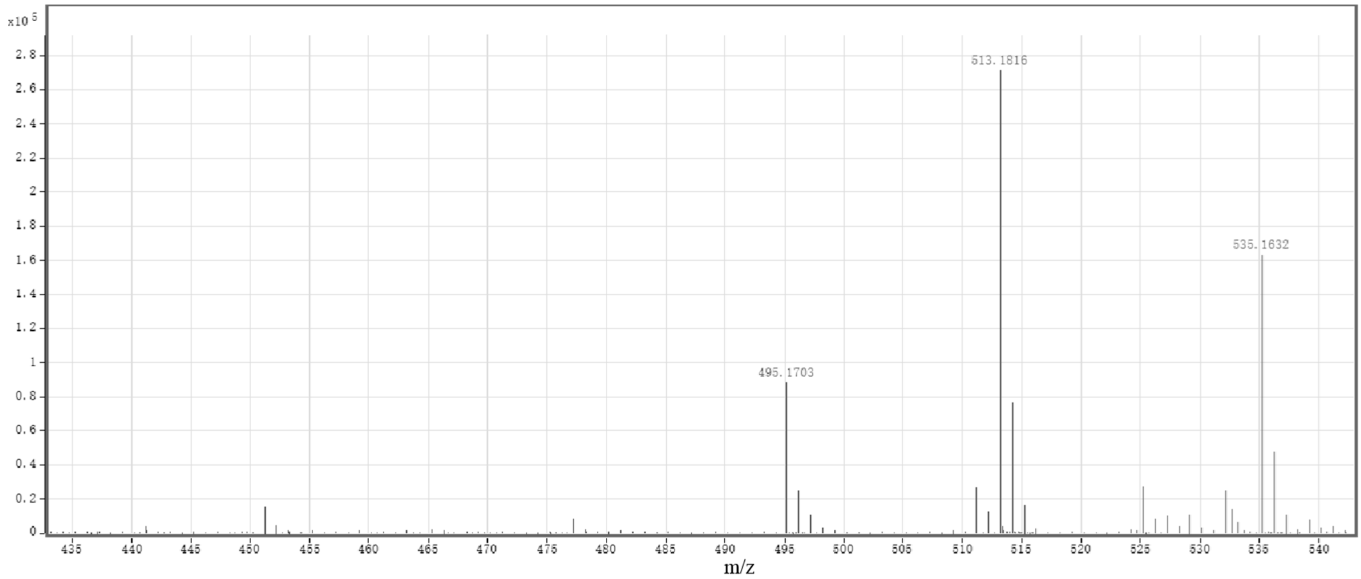

Figure S2a. HRESIMS spectrum of **2**

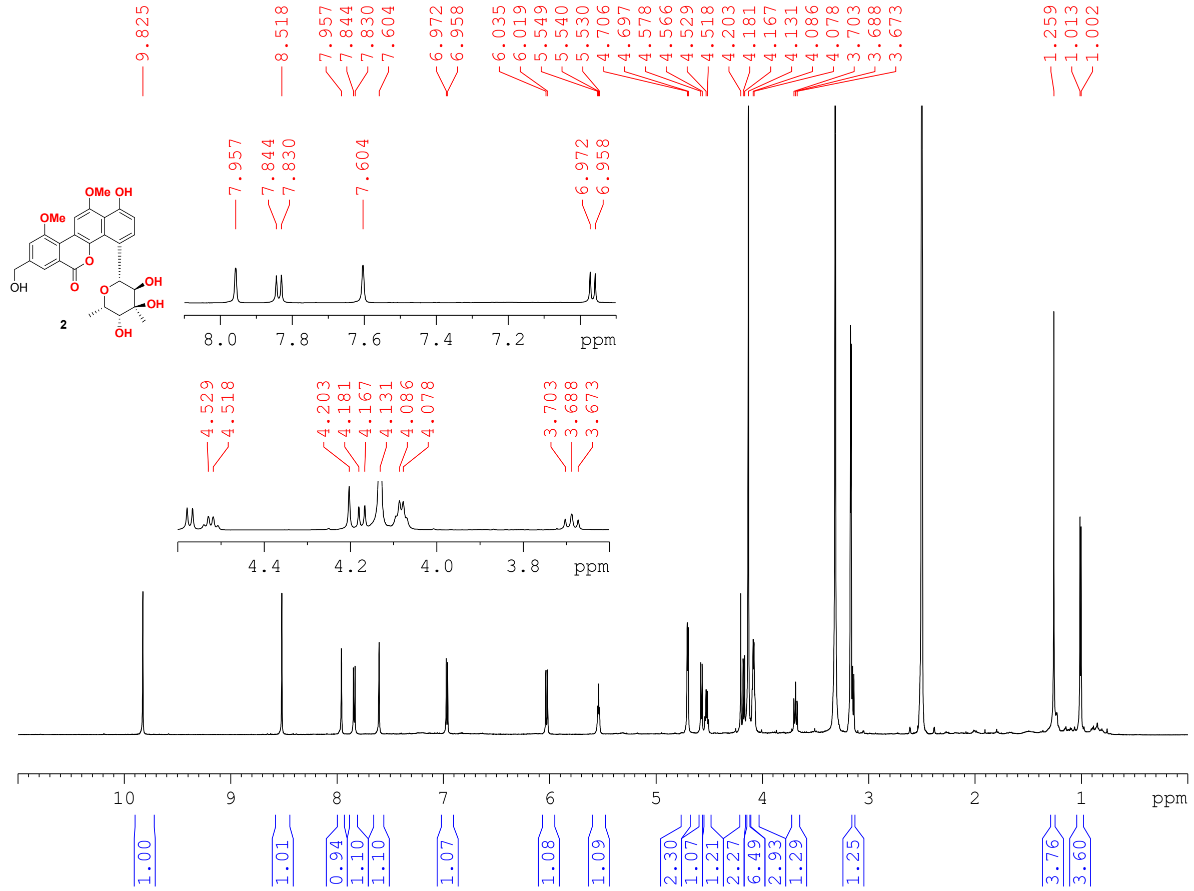

Figure S2b. ¹H NMR spectrum (600 MHz, DMSO-d_6) of **2**

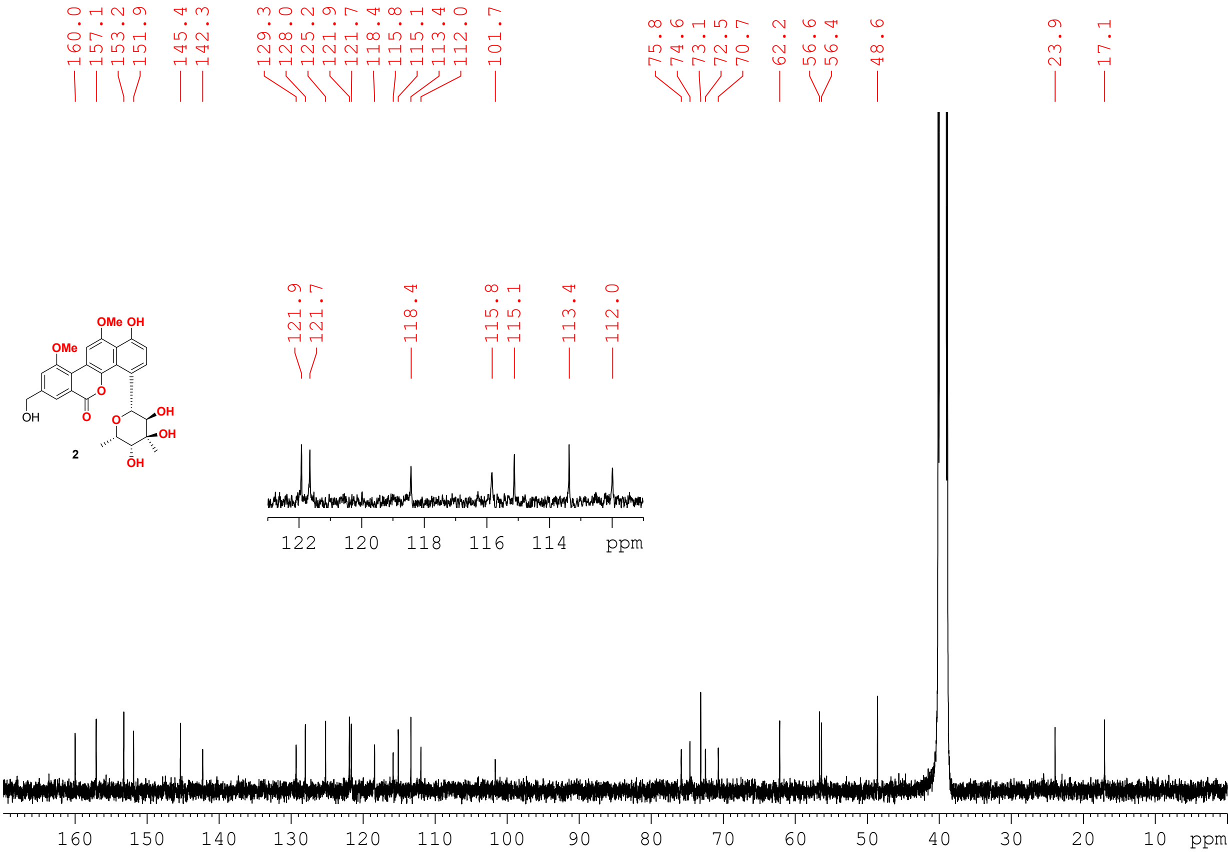

Figure S2c. ^{13}C NMR spectrum (150 MHz, DMSO-d_6) of **2**

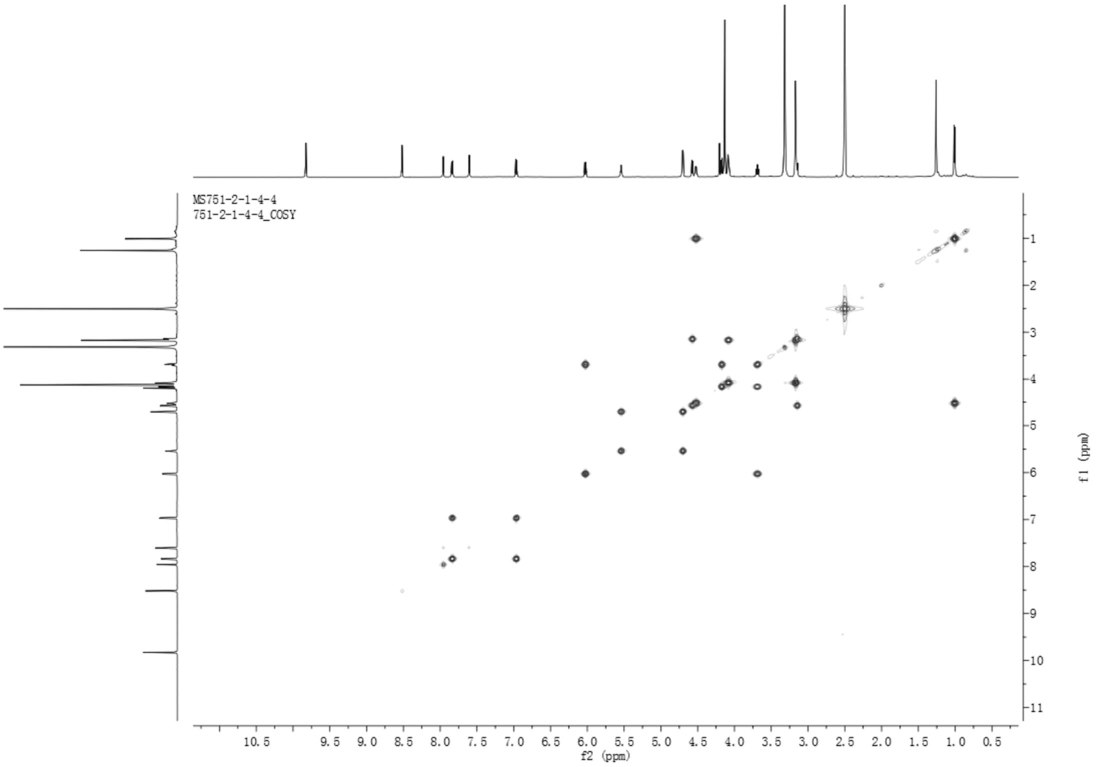

Figure S2d. COSY spectrum (600 MHz, DMSO-d_6) of **2**

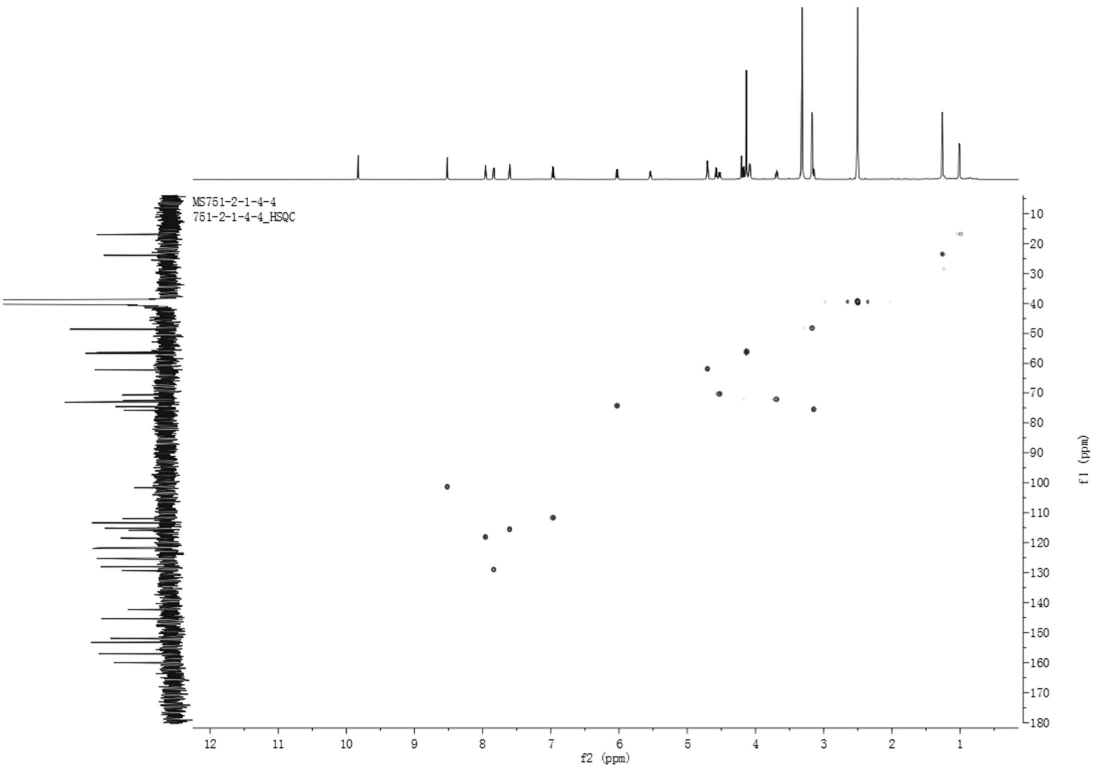

Figure S2e. ^{1}H – ^{13}C HSQC spectrum (600 MHz, DMSO-d_6) of **2**

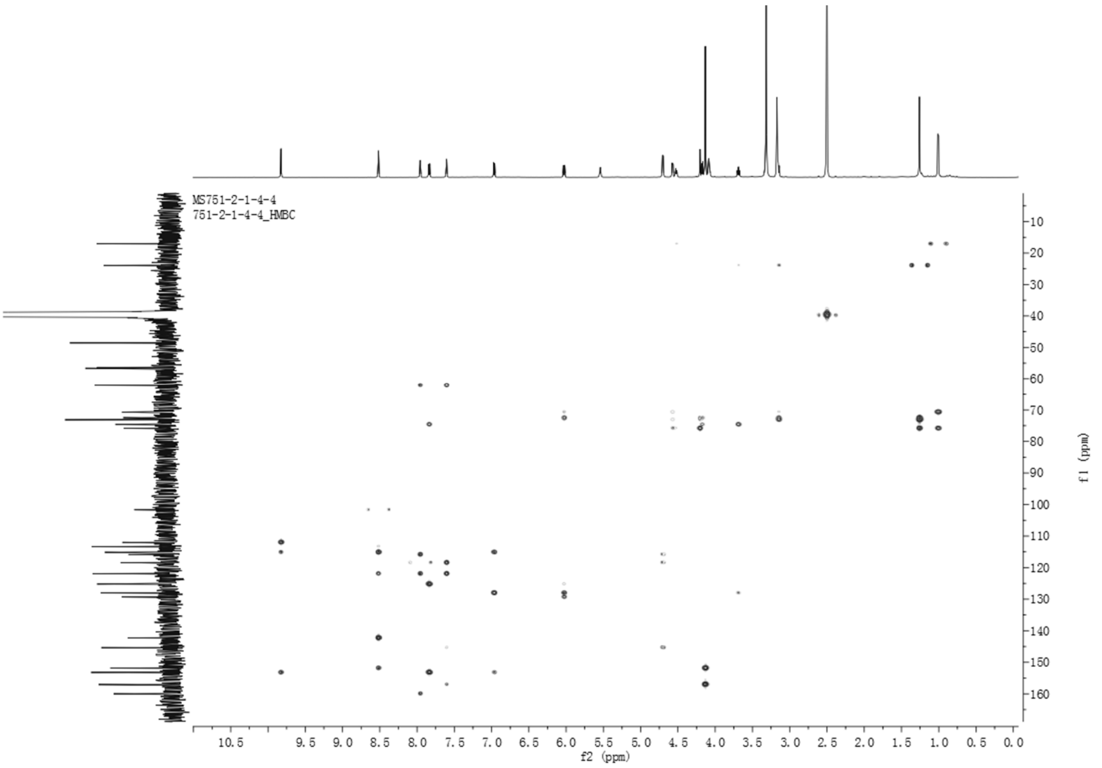

Figure S2f. ^{1}H – ^{13}C HMBC spectrum (600 MHz, DMSO-d_6) of **2**

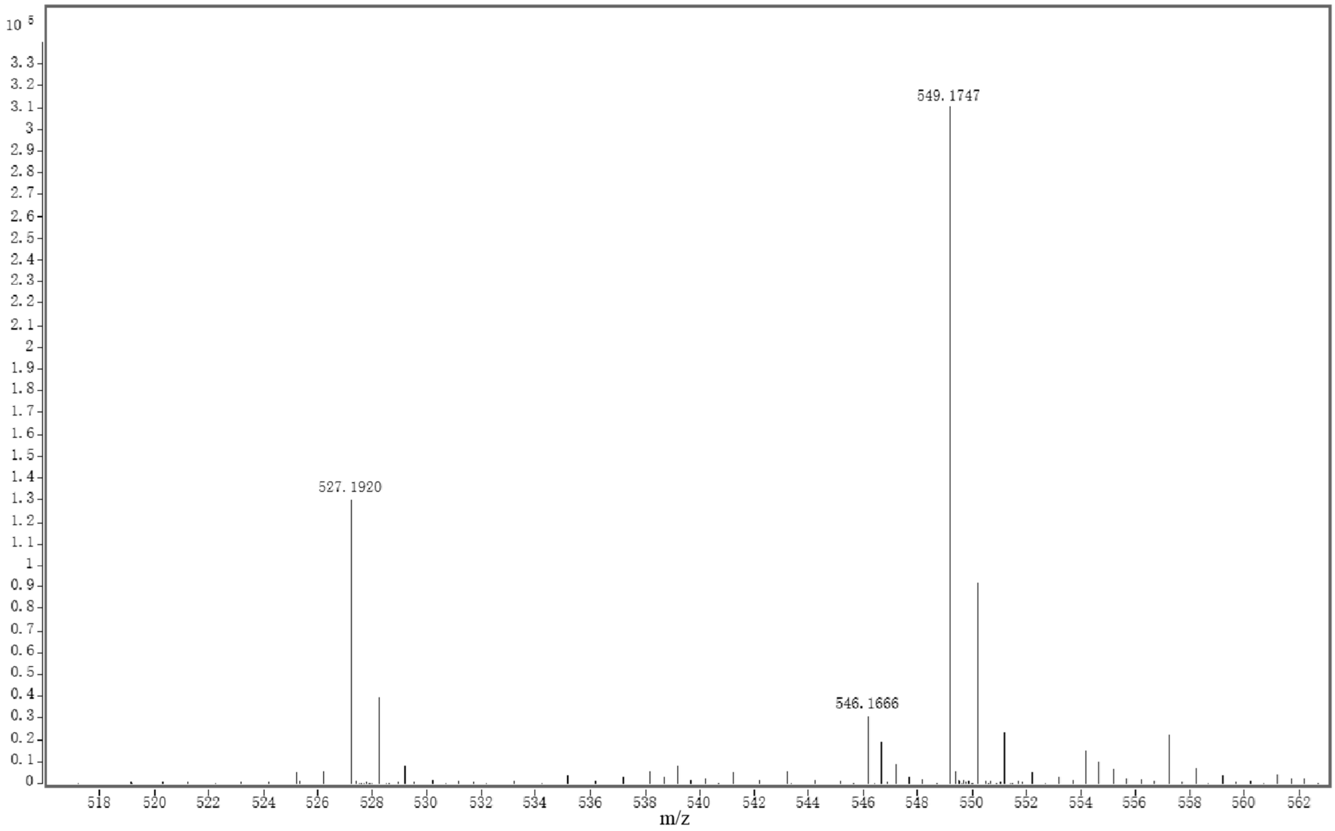

Figure S3a. HRESIMS spectrum of **3**

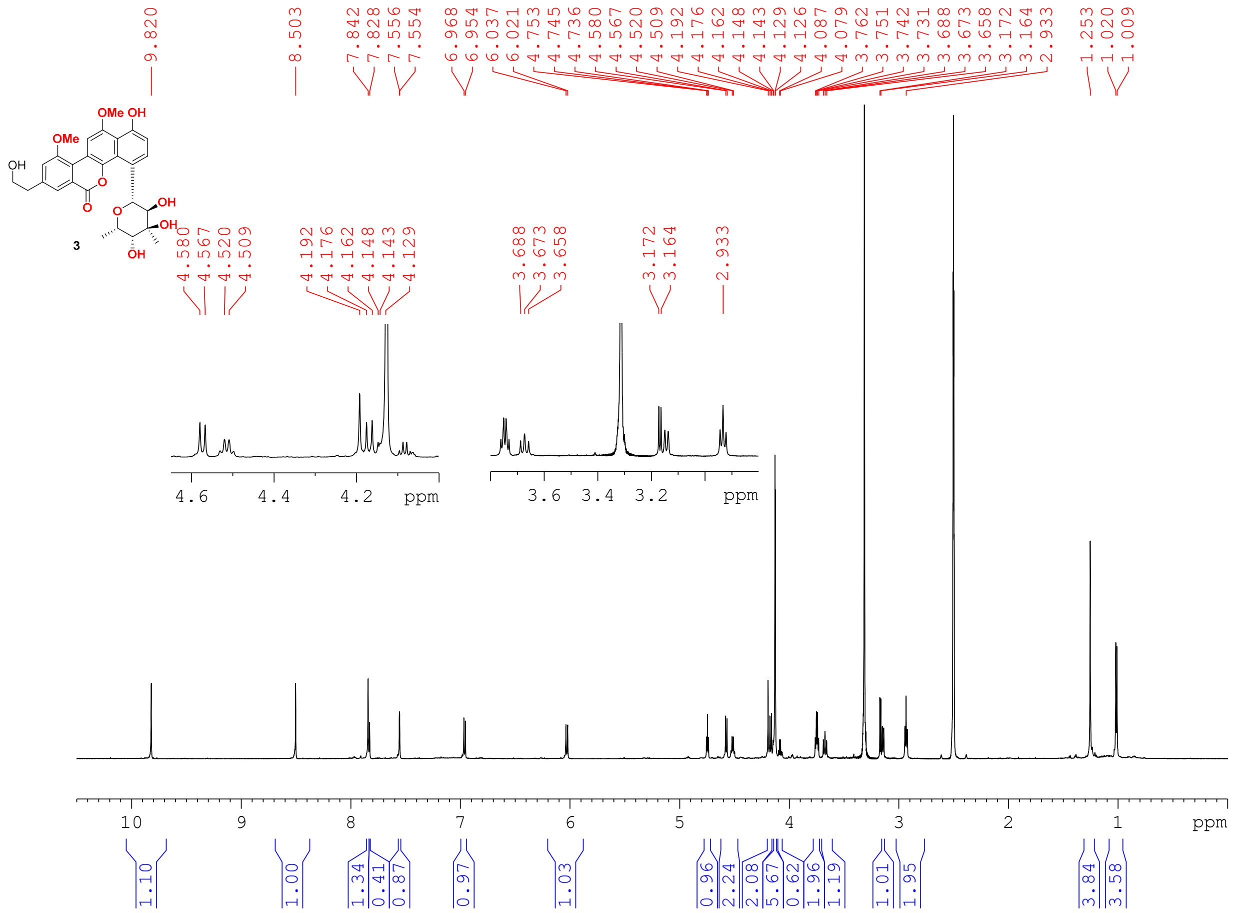

Figure S3b. ^{1}H NMR spectrum (600 MHz, DMSO-d_6) of **3**

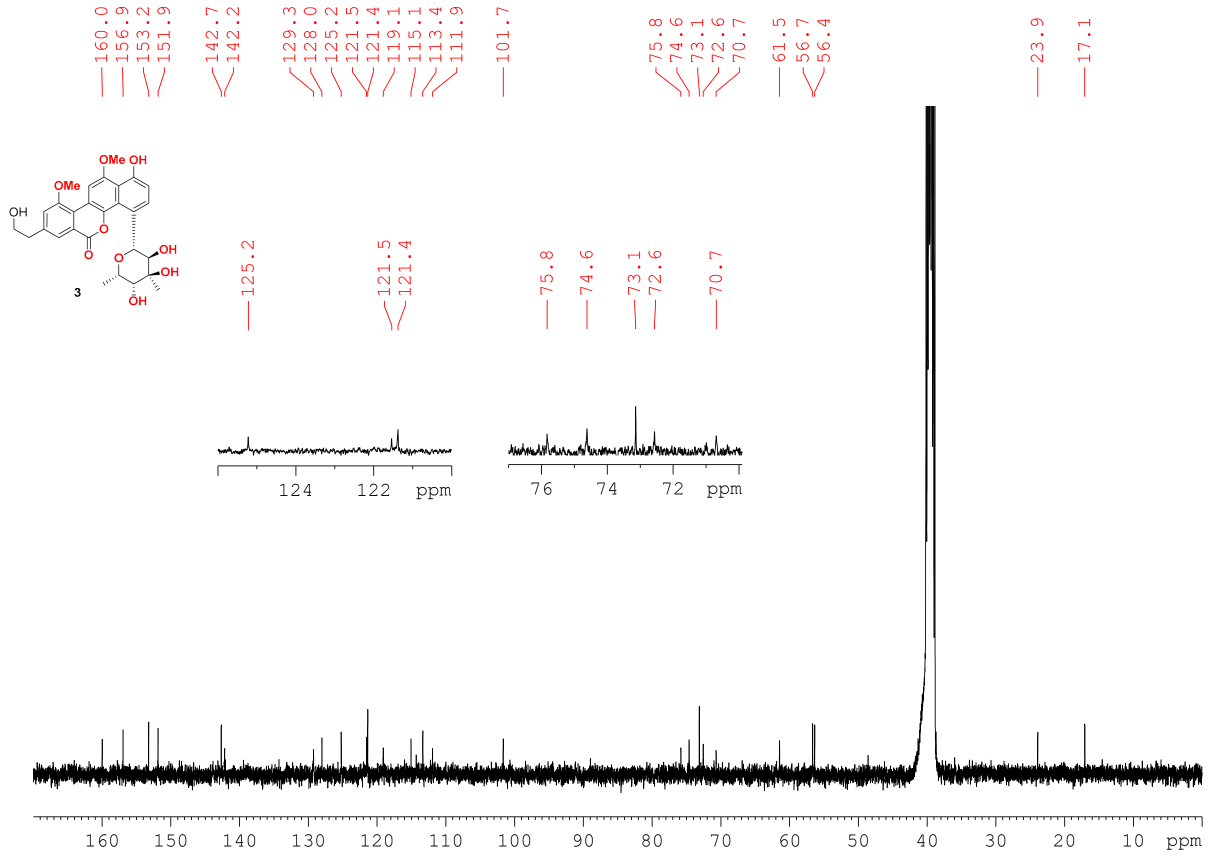

Figure S3c. ^{13}C NMR spectrum (150 MHz, DMSO-d_6) of **3**

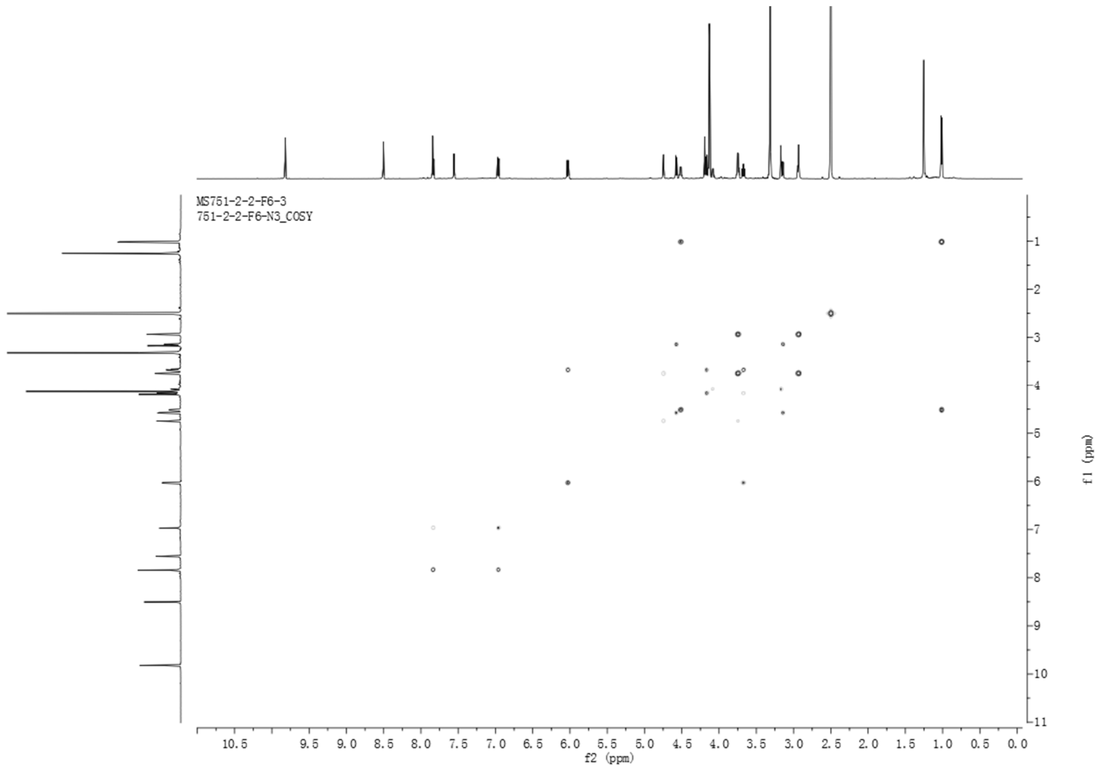

Figure S3d. COSY spectrum (600 MHz, DMSO-d_6) of **3**

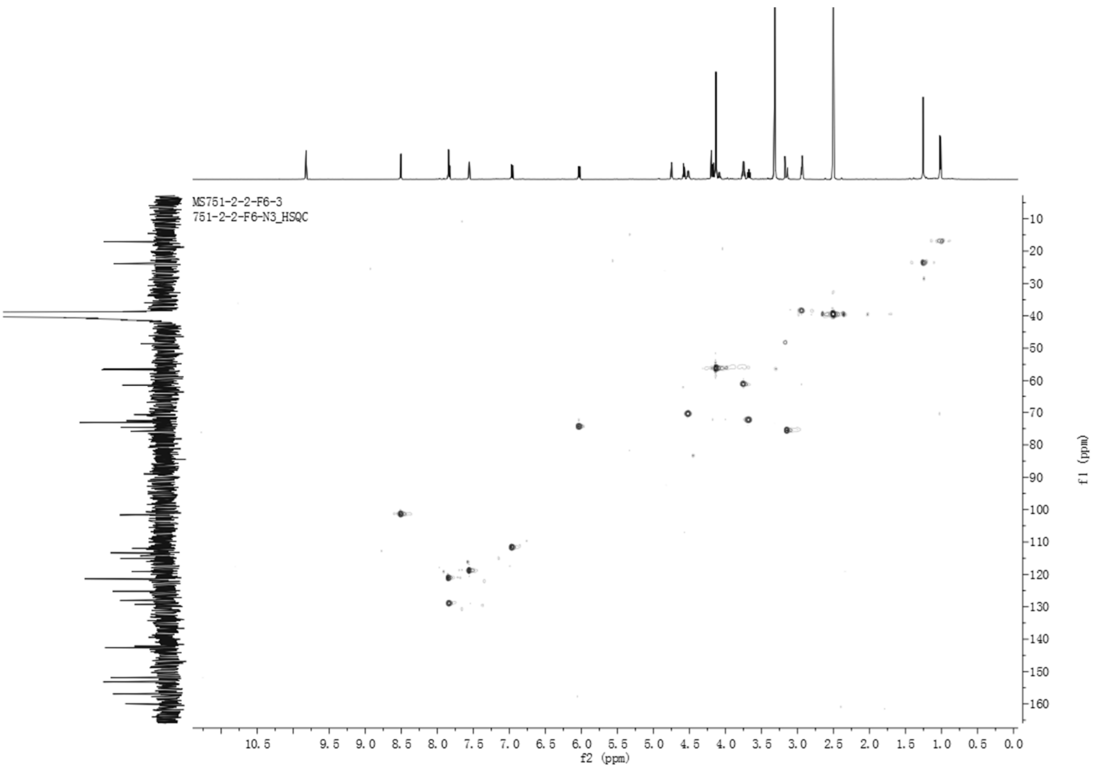

Figure S3e. ^{1}H – ^{13}C HSQC spectrum (600 MHz, DMSO-d_6) of **3**

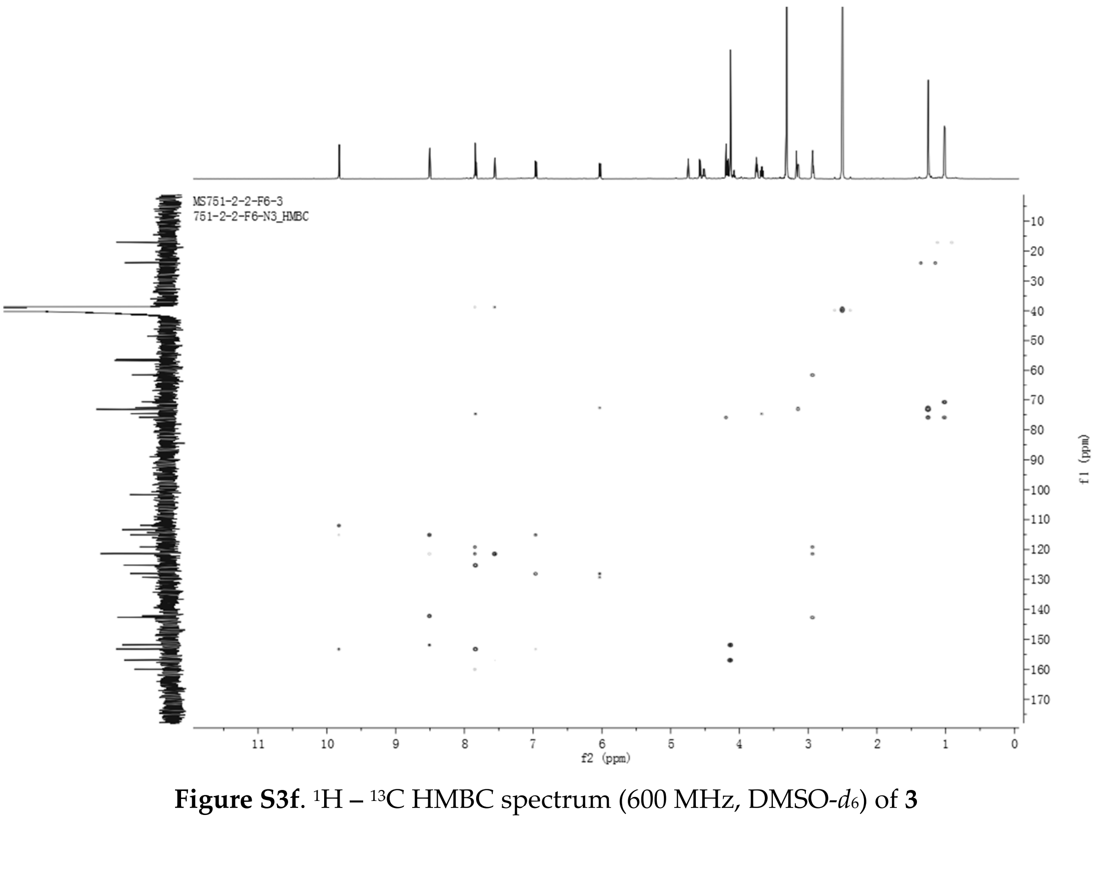

Figure S3f. ^{1}H – ^{13}C HMBC spectrum (600 MHz, DMSO-d_6) of **3**

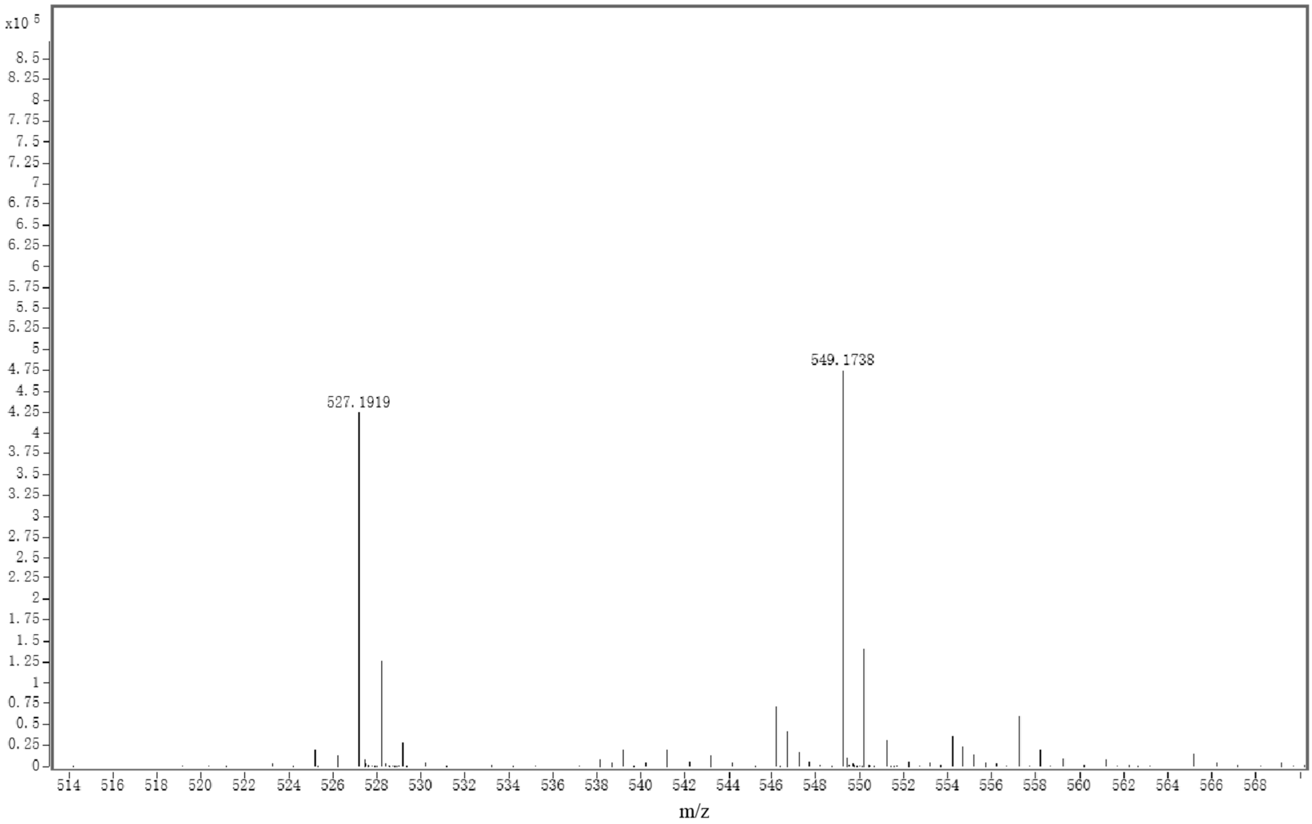

Figure S4a. HRESIMS spectrum of **4**

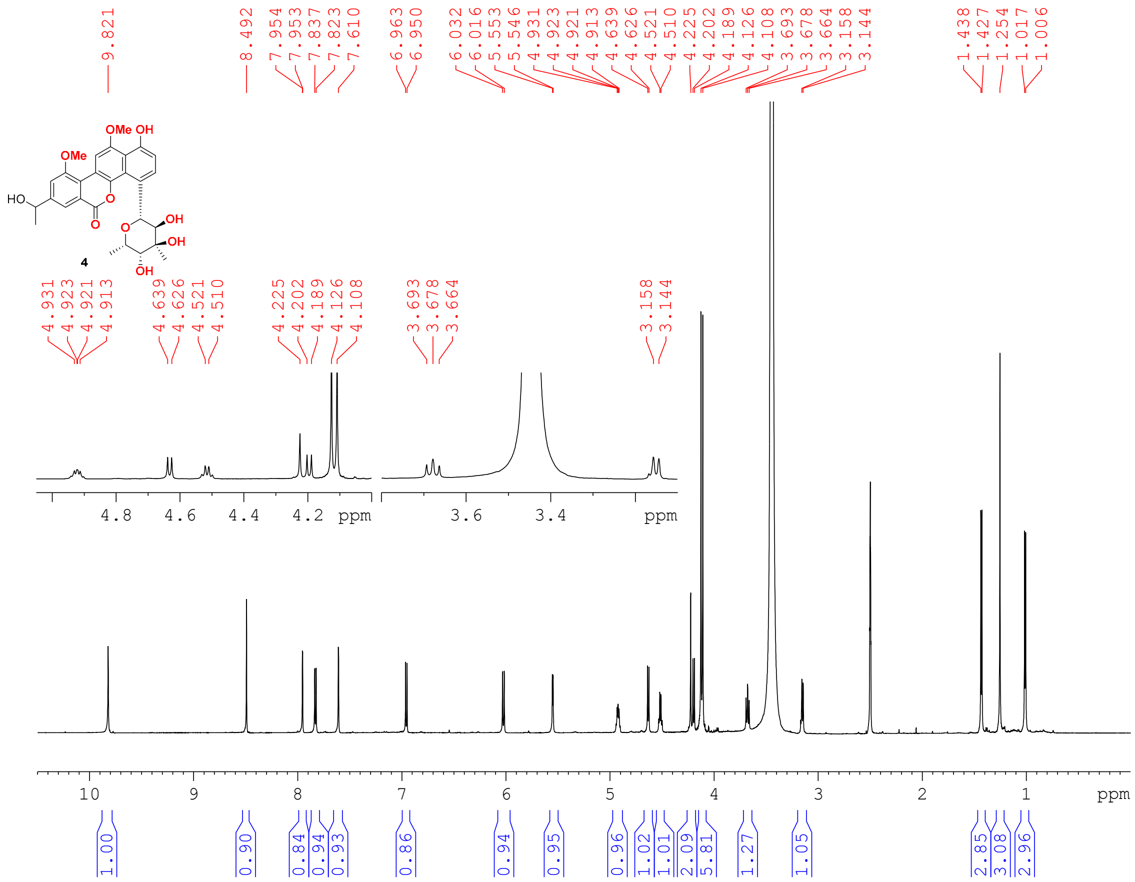

Figure S4b. ^{1}H NMR spectrum (600 MHz, DMSO-d_6) of **4**

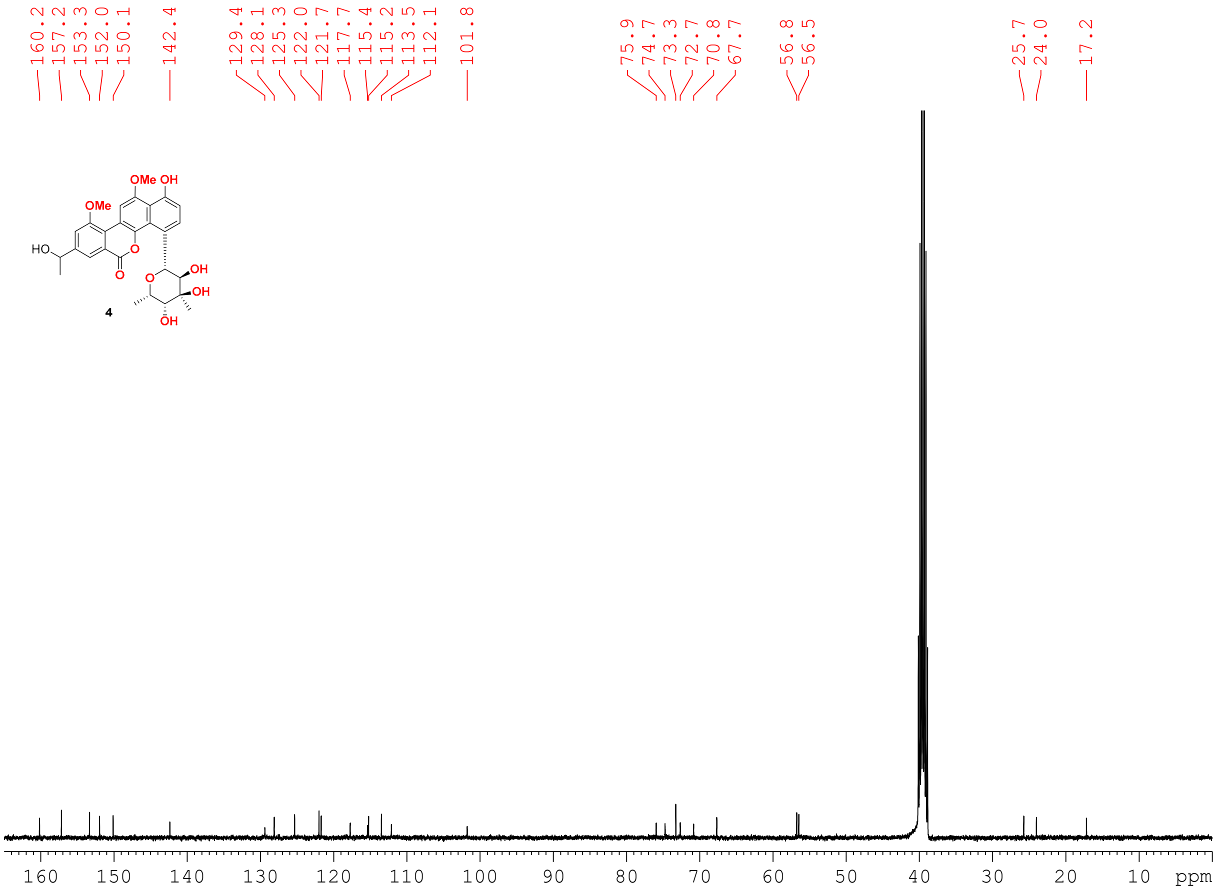

Figure S4c. ^{13}C NMR spectrum (150 MHz, DMSO-d_6) of **4**

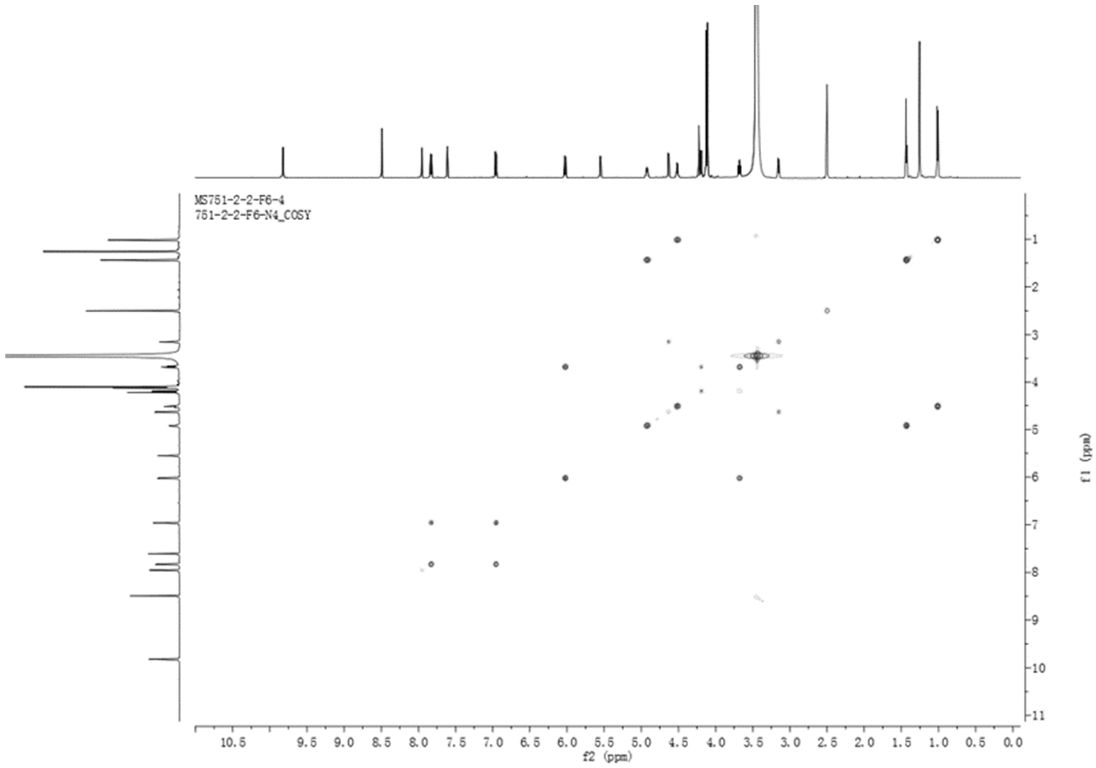

Figure S4d. COSY spectrum (600 MHz, DMSO-d_6) of 4

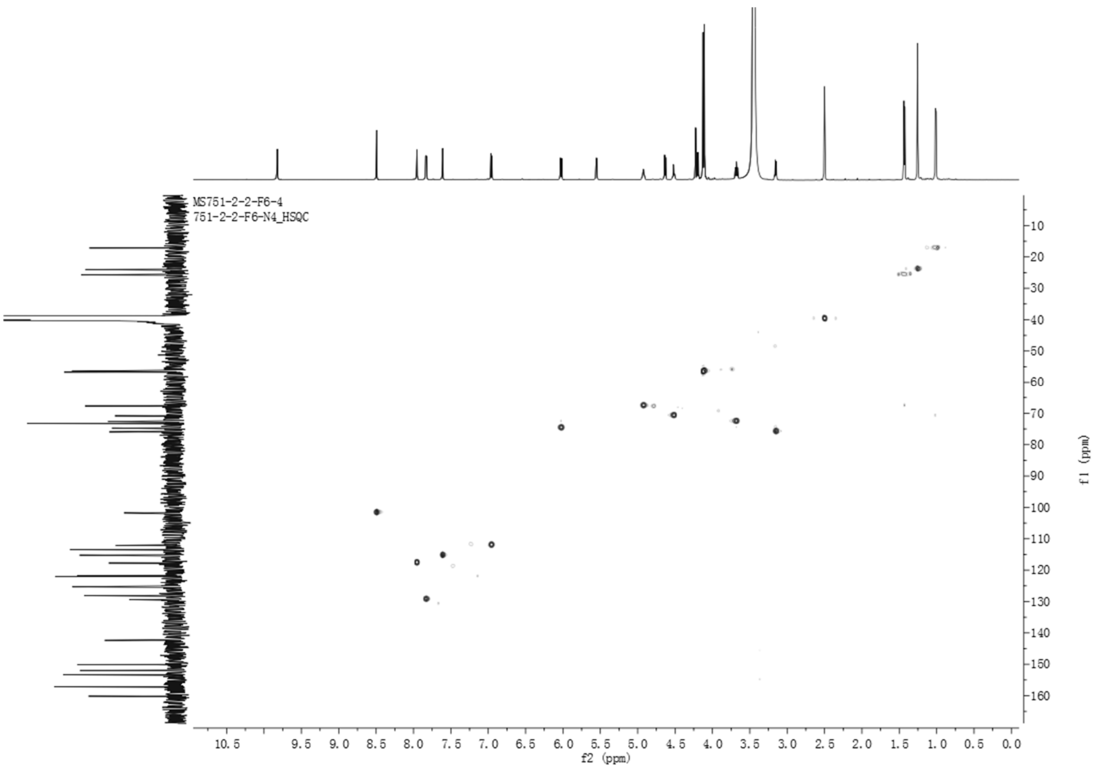

Figure S4e. ^1H – ^{13}C HSQC spectrum (600 MHz, DMSO-d_6) of 4

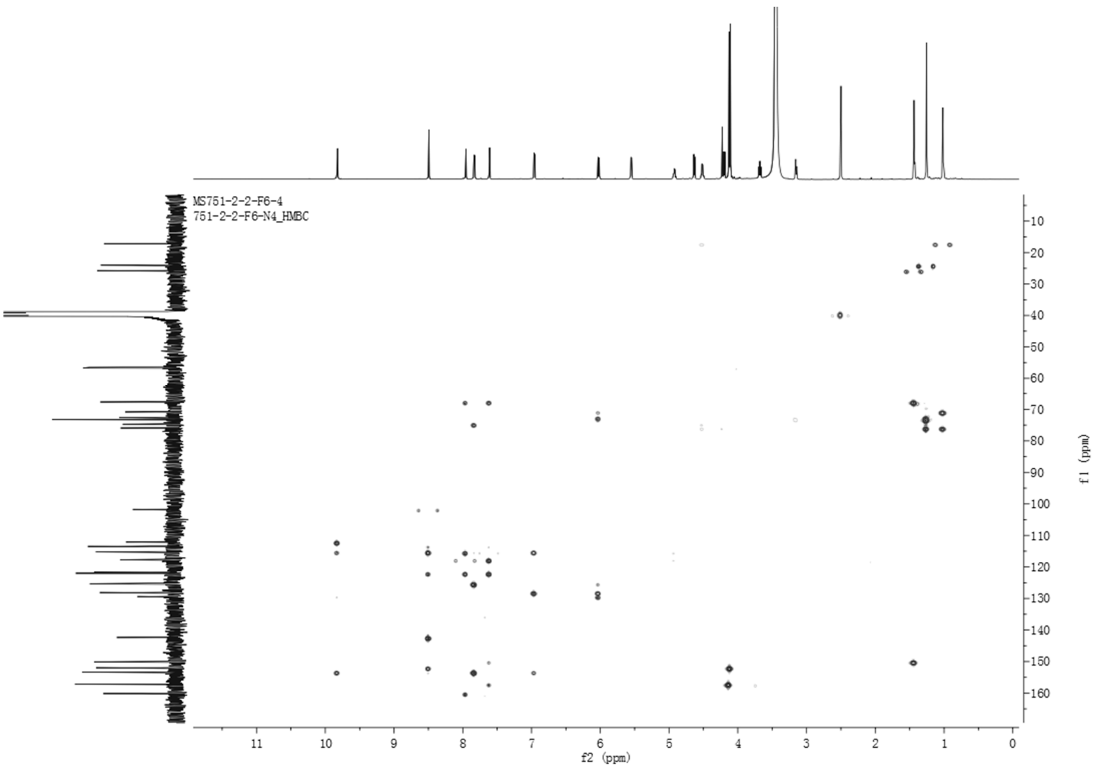

Figure S4f. ^1H – ^{13}C HMBC spectrum (600 MHz, DMSO-d_6) of 4

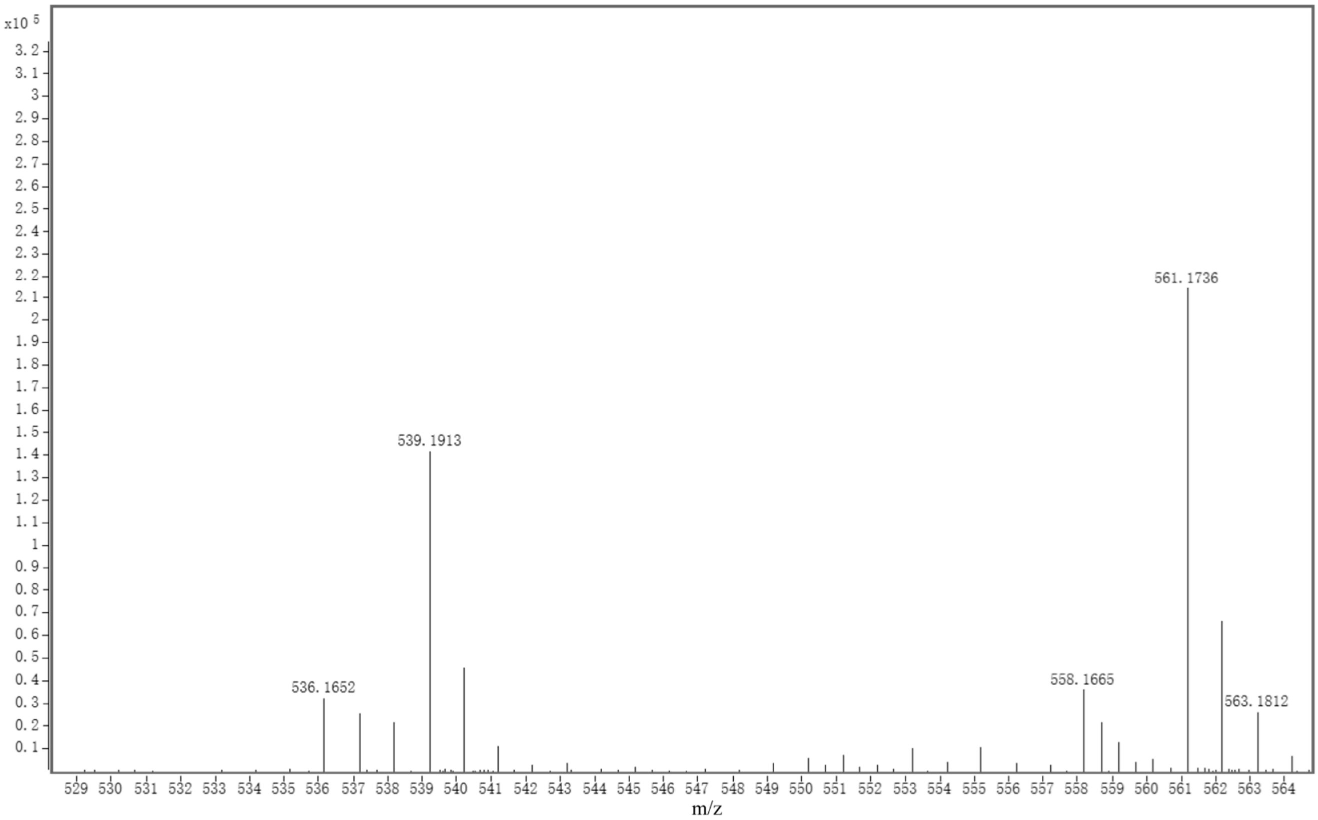

Figure S5a. HRESIMS spectrum of **5**

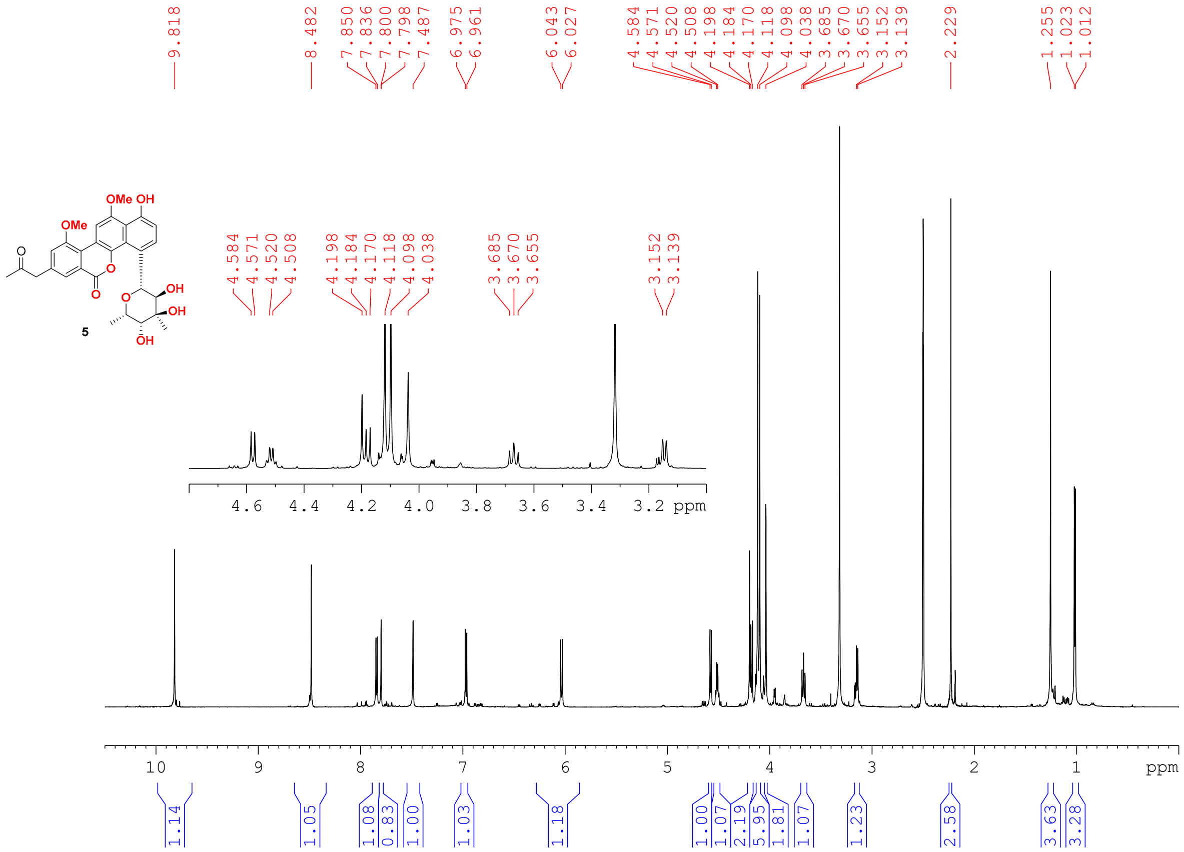

Figure S5b. ^{1}H NMR spectrum (600 MHz, DMSO-*d*6) of **5**

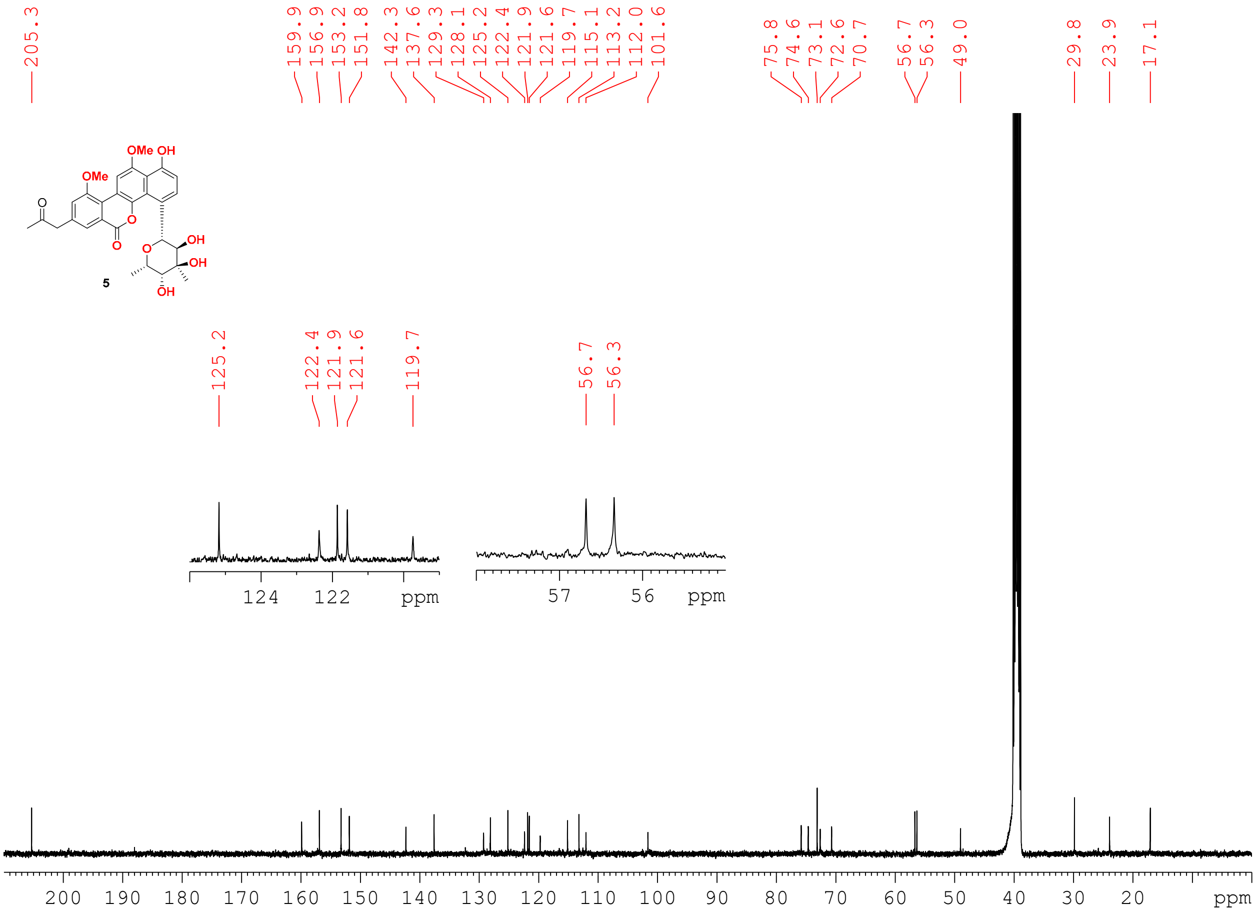

Figure S5c. ^{13}C NMR spectrum (150 MHz, DMSO-d_6) of **5**

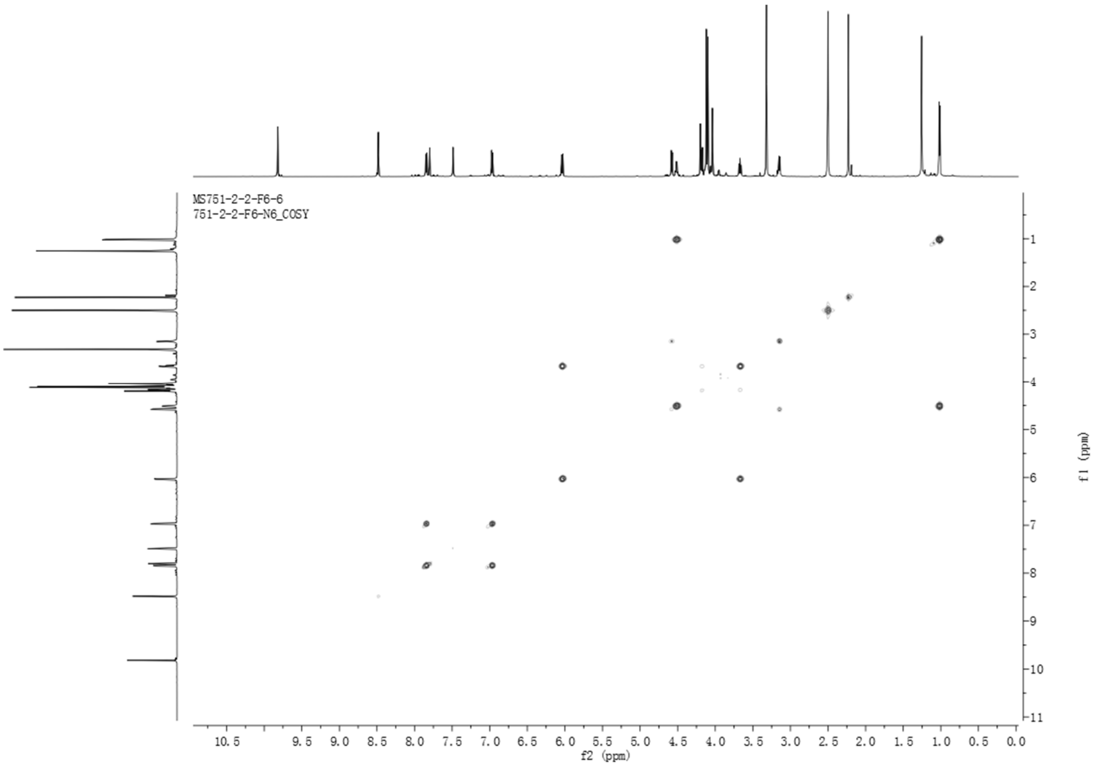

Figure S5d. COSY spectrum (600 MHz, DMSO-d_6) of **5**

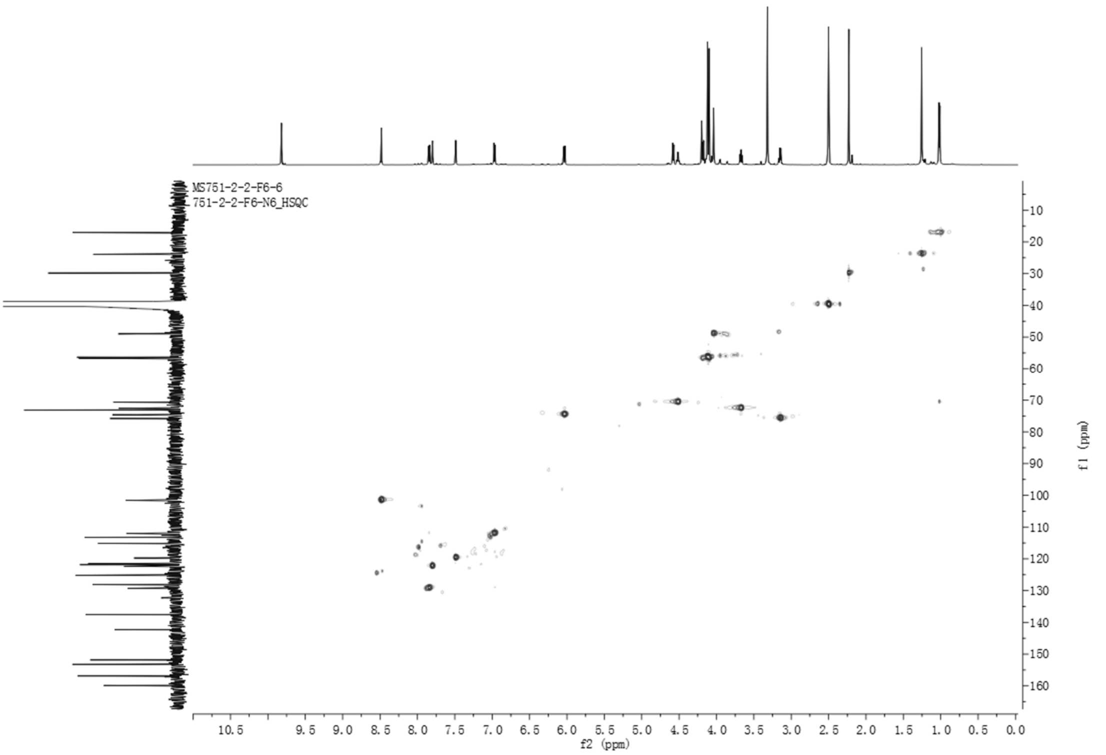

Figure S5e. ^{1}H – ^{13}C HSQC spectrum (600 MHz, DMSO-d_6) of **5**

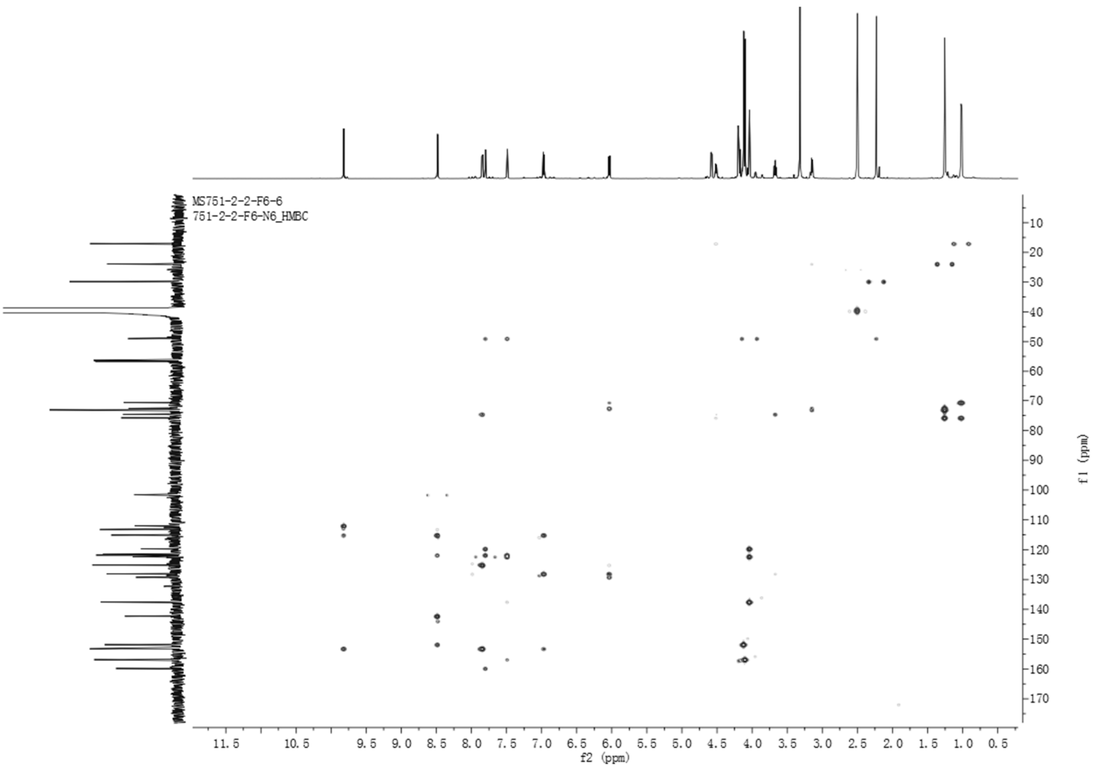

Figure S5f. ^{1}H – ^{13}C HMBC spectrum (600 MHz, DMSO-d_6) of **5**

Table S1. Screening of conditions for the [2+2] photodimerization of chrysomycin A

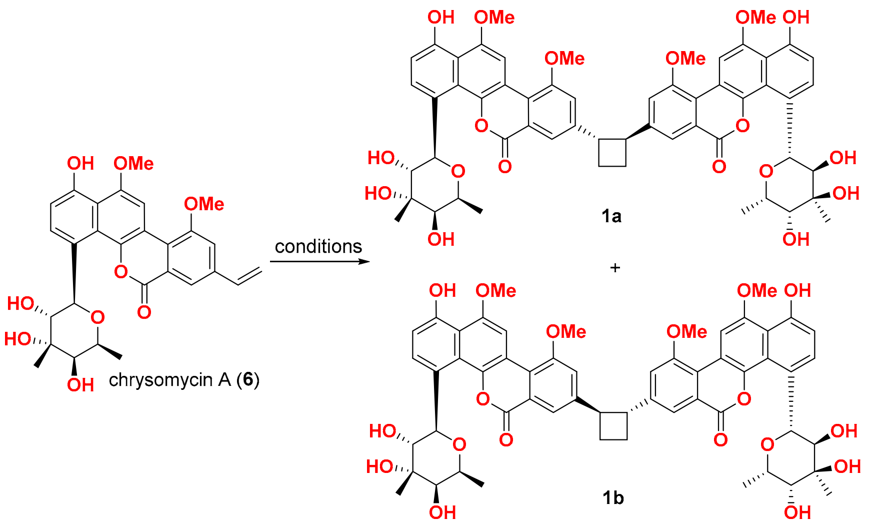

entry	conditions	yield[a,b]
1	neat, sunlight, r.t. 2 h	decomp.
2	neat, 500 W high-pressure Hg lamp, 0 °C, 40 min	NR
3	$CDCl_3$, 500 W high-pressure Hg lamp, r.t. 1.5 h	decomp.
4	hexane, 500 W high-pressure Hg lamp, r.t. 1 h	NR
5	9-cyanoanthracene, CH_3CN, 500 W high-pressure Hg lamp, 2 h	NR
**6**	**CH_2Cl_2, 11 W CFL, sealed tube, 75-80 °C, 60 h**	**40% (1:1)**

[a]Combined yield of **1a** and **1b**. [b]Ratio of **1a**:**1b** is given in the parenthesis. CFL = compact fluorescent light bulb. NR = no reaction.

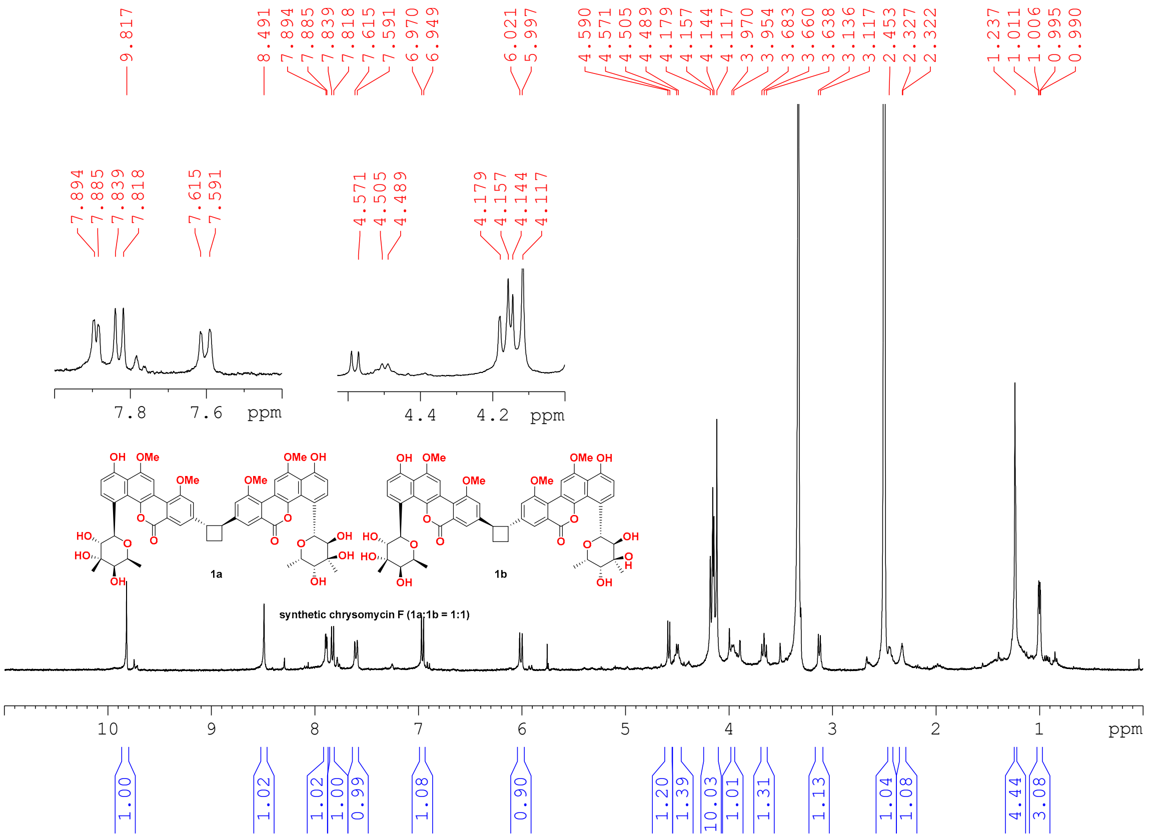

Figure S6a. ^{1}H NMR spectrum (400 MHz, DMSO-d_6) of synthetic chrysomycin F (**1**)

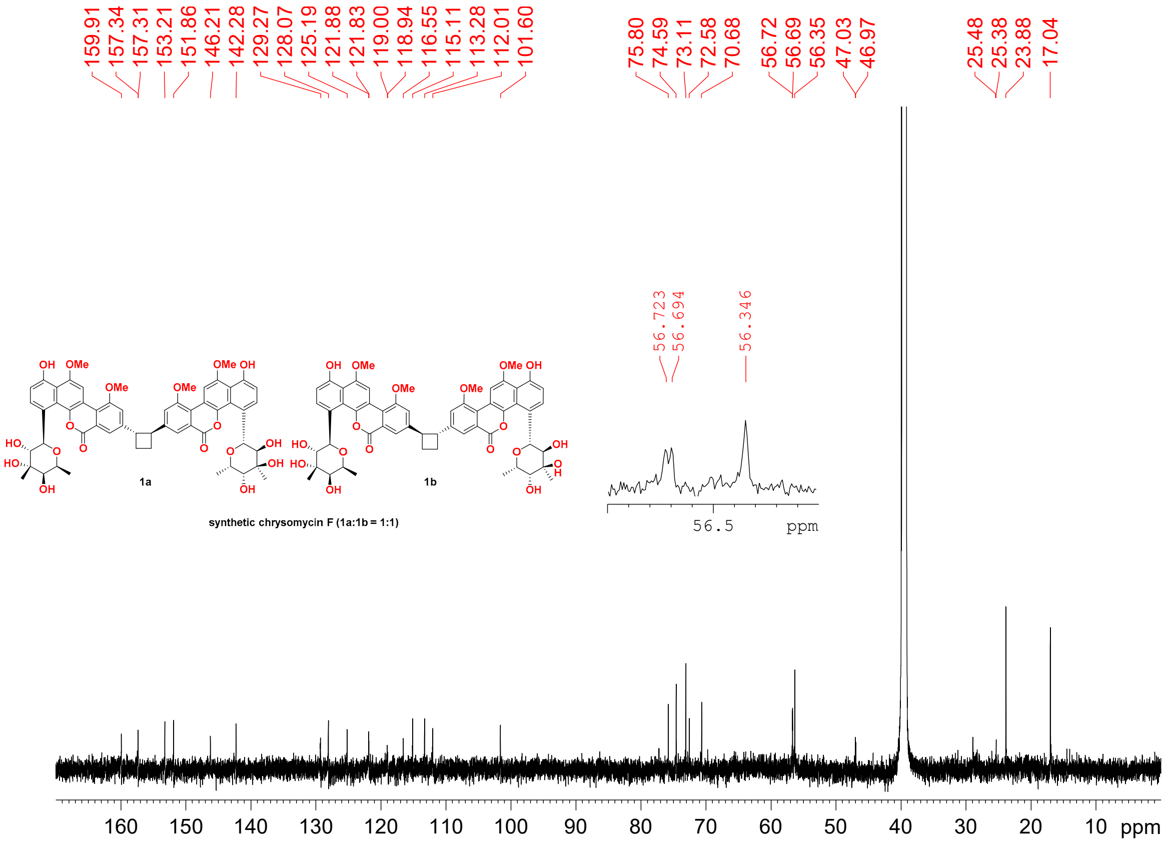

Figure S6b. ^{13}C NMR spectrum (201 MHz, DMSO-d_6) of synthetic chrysomycin F (**1**)

Table S2. ^{1}H and ^{13}C NMR data comparison of natural and synthetic chrysomycin F (**1**) in DMSO-d_6

	natural chrysomycin F		synthetic chrysomycin F	
**position**	**δ_H,[a] *mult* (*J* in Hz)**	**δ_C[b]**	**δ_H,[c] *mult* (*J* in Hz)**	**δ_C[d]**
1/1'		153.2, C		153.2, C
2/2'	6.96, d (*8.4*)	112.0, CH	6.96, d (*8.4*)	112.0, CH
3/3'	7.83, d (*8.4*)	129.3, CH	7.83, d (*8.4*)	129.3, CH
4/4'		128.1, C		128.1, C
4a/4a'		125.2, C		125.2, C
4b/4b'		142.3, C		142.3, C
6/6'		159.9, C		159.9, C
6a/6a'		121.9, C		121.9, C
7/7'	7.88/7.89, d (*1.2*)	119.0/118.9, CH	7.88/7.89, d (*1.2*)	119.0/118.9, CH
8/8'		146.2, C		146.2, C
9/9'	7.58/7.61, d(*1.2*)	116.5, CH	7.59/7.61, d(*1.2*)	116.5, CH

10/10'		157.35/157.32, C		157.34/157.31, C
10a/10a'		121.8, C		121.8, C
10b/10b'		113.3, C		113.3, C
11/11'	8.49, s	101.6, CH	8.49, s	101.6, CH
12/12'		151.9, C		151.9, C
12a/12a'		115.1, C		115.1, C
13/13'	6.01, d (*9.6*)	74.6, CH	6.01, d (*9.6*)	74.6, CH
14/14'	3.66, dd (*9.6, 8.4*)	72.6, CH	3.66, t (*8.9*)	72.6, CH
15/15'		73.1, C		73.1, C
16/16'	3.13, d (*7.8*)	75.8, CH	3.13, d (*7.7*)	75.8, CH
17/ 17'	4.50, brq (*6.0*)	70.7, CH	4.50, brq (*6.4*)	70.7, CH
18/18'	3.96, m	47.05/46.99, CH	3.96, m	47.03/46.97, CH
19a/19'a	2.33, m	25.4/25.5, CH_2	2.33, m	25.4/25.5, CH_2
19b/19'b	2.45, m		2.45, m	
20/20'	4.15/4.14, s	56.74/56.71, CH_3	4.16/4.14, s	56.72/56.69, CH_3
21/21'	4.11, s	56.3, CH_3	4.12, s	56.3, CH_3
22/22'	1.24, s	23.9, CH_3	1.24, s	23.9, CH_3
23/23'	1.00/1.01, d (*6.6*)	17.1, CH_3	1.00/1.01, d, (*6.5*)	17.0, CH_3
1/1'-OH	9.81, s		9.82, s	
14/14'-OH	4.16, overlap (*8.4*)		4.17 overlap (*8.8*)	
15/15'-OH	4.18, s		4.18, s	
16/16'-OH	4.57, d (*7.8*)		4.58, d (*7.7*)	

[a] ^1H (600 MHz), [b] ^{13}C (150 MHz), [c] ^1H (400 MHz), [d] ^{13}C (201 MHz)

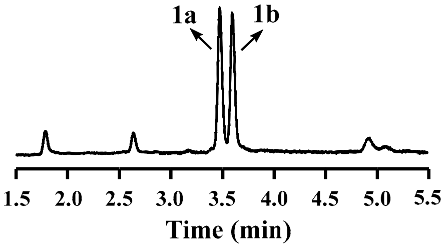

Figure S7. UPLC chromatogram of synthetic chrysomycin F (**1**).

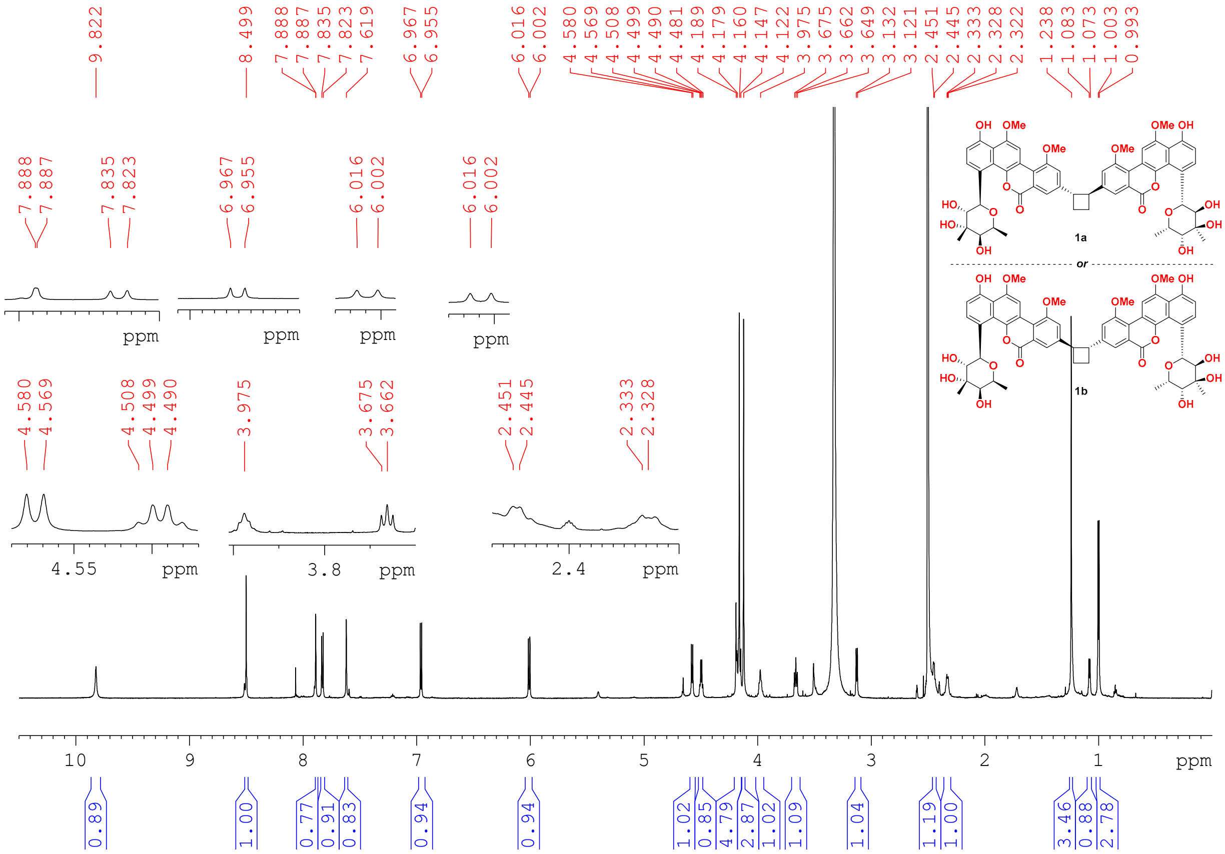

Figure S8a. ^{1}H NMR spectrum (700 MHz, DMSO-*d*$_6$) of synthetic *trans*-dimer **1a** (or **1b**)

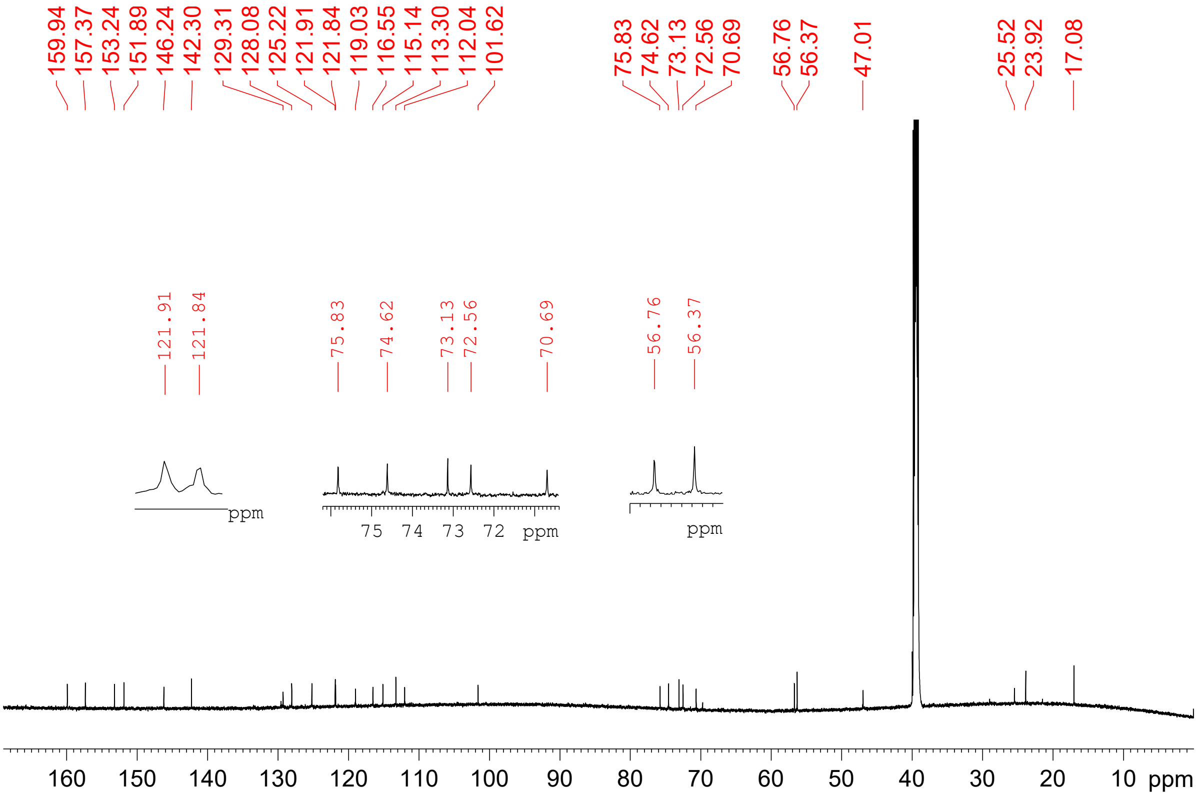

Figure S8b. ^{13}C NMR spectrum (176 MHz, DMSO-d_6) of synthetic *trans*-dimer **1a** (or **1b**)

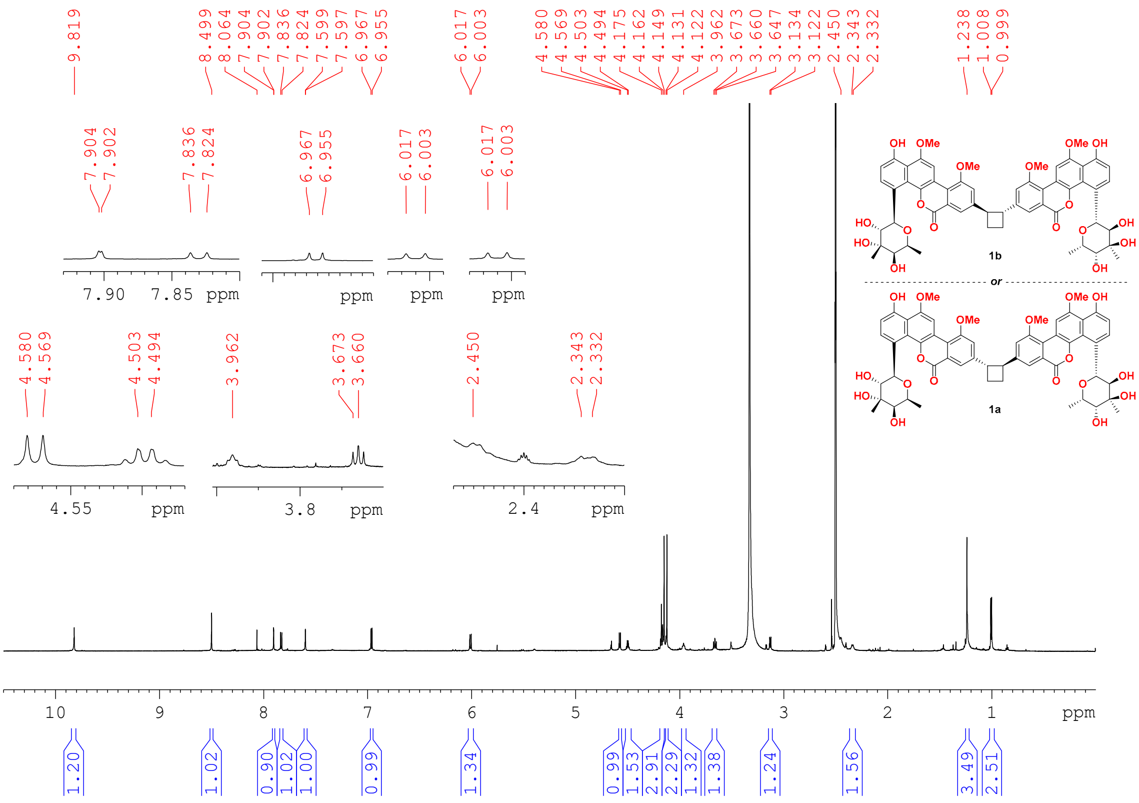

Figure S9a. ^{1}H NMR spectrum (700 MHz, DMSO-d_6) of synthetic *trans*-dimer **1b** (or **1a**)

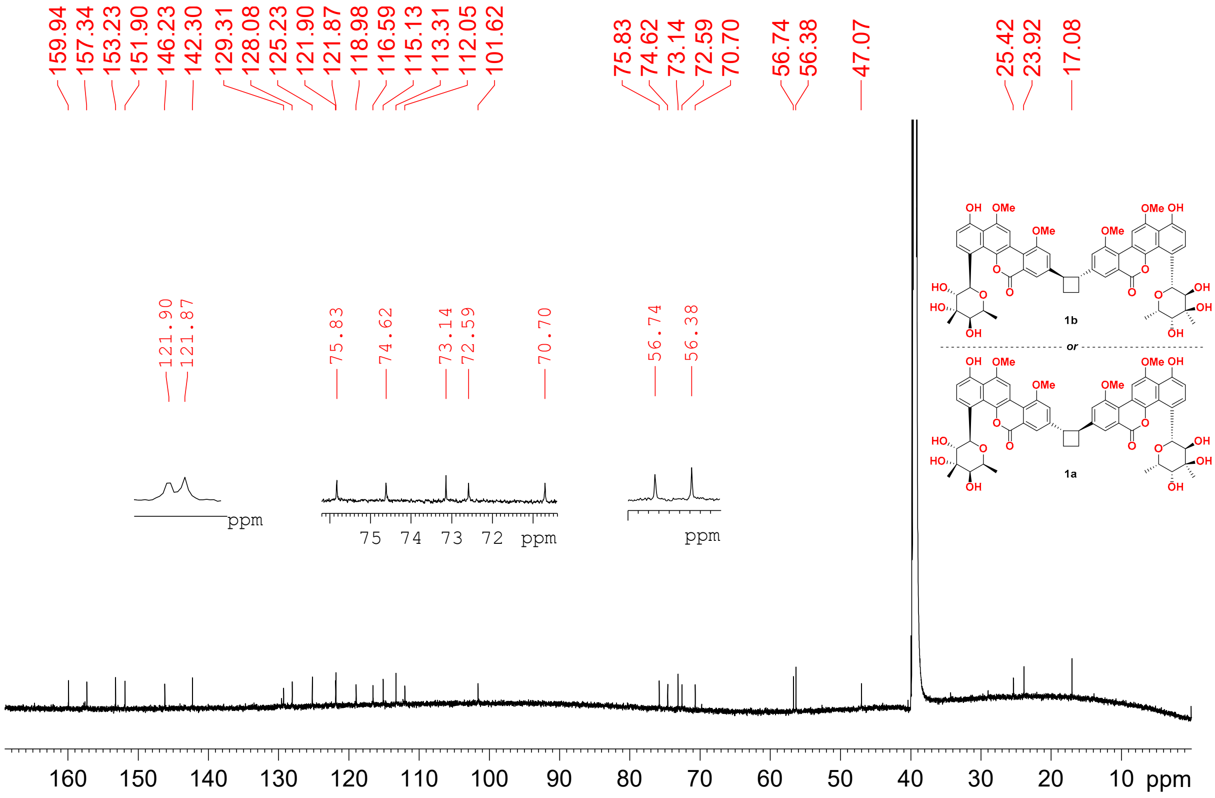

Figure S9b. ^{13}C NMR spectrum (176 MHz, DMSO-d_6) of synthetic *trans*-dimer **1b** (or **1a**)

Table S3. ^{1}H and ^{13}C NMR data for synthetic *trans*-dimers **1a** and **1b** in DMSO-d_6

	trans-dimer 1a		*trans*-dimer 1b	
**position**	**δ_H,[a] *mult* (*J* in Hz)**	**δ_C[b]**	**δ_H,[a] *mult* (*J* in Hz)**	**δ_C[b]**
1/1'		153.24, C		153.23, C
2/2'	6.96, d (*8.4*)	112.04, CH	6.96, d (*8.4*)	112.05, CH
3/3'	7.83, d (*8.4*)	129.31, CH	7.83, d (*8.4*)	129.31, CH
4/4'		128.08, C		128.08, C
4a/4a'		125.22, C		125.23, C
4b/4b'		142.30, C		142.30, C
6/6'		159.94, C		159.94, C
6a/6a'		121.91, C		121.90, C
7/7'	7.89, d (*0.7*)	119.03, CH	7.90, d (*1.4*)	118.98, CH
8/8'		146.24, CH		146.23, C
9/9'	7.62, d (*0.7*)	116.55, CH	7.60, d (*1.4*)	116.59, CH
10/10'		157.37, C		157.34, C
10a/10a'		121.84, C		121.87, C
10b/10b'		113.30, C		113.31, C
11/11'	8.50, s	101.62, CH	8.50, s	101.62, CH
12/12'		151.89, C		151.90, C
12a/12a'		115.14, C		115.13, C
13/13'	6.01, d (*9.8*)	74.62, CH	6.01, d (*9.8*)	74.62, CH
14/14'	3.66, dd (*9.8, 9.1*)	72.56, CH	3.66, dd (*9.8, 9.1*)	72.59, CH
15/15'		73.13, C		73.14, C
16/16'	3.13, d (*7.7*)	75.83, CH	3.13, d (*8.4*)	75.83, CH
17/ 17'	4.49, q (*7.0*)	70.69, CH	4.50, q (*7.0*)	70.70, CH
18/18'	3.98, m	47.01, CH	3.96, m	47.07, CH
19a/19'a	2.33, m	25.52, CH_2	2.34, m	25.42, CH_2
19b/19'b	2.45, m		2.45, m	

20/20'	4.16, s	56.76, CH_3	4.15, s	56.74, CH_3
21/21'	4.12, s	56.37, CH_3	4.12, s	56.38, CH_3
22/22'	1.24, s	23.92, CH_3	1.24, s	23.92, CH_3
23/23'	1.00, d, (*7.0*)	17.08, CH_3	1.00, d, (*7.0*)	17.08, CH_3
1/1'-OH	9.82, s		9.82, s	
14/14'-OH	4.17 overlap (*9.1*)		4.17 overlap (*9.1*)	
15/15'-OH	4.19, s		4.17, s	
16/16'-OH	4.57, d (*7.7*)		4.57, d (*7.7*)	

[a] ^{1}H (700 MHz), [b] ^{13}C (176 MHz)

Table S4. Composition of the cluture media.

Media	Component
Gauze–asparagine (GA) agar plate	soluble starch 2.0 %, L-asparagine 0.05 %, KNO_3 0.1 %, $K_2HPO_4·H_2O$ 0.05 %, NaCl 0.05 %, and $MgSO_4·7H_2O$ 0.05 % (pH 7.5)
ISP2 liquid medium	yeast extract 0.4%, malt extract 1%, glucose 0.4%, pH 7.2
AM2 liquid medium	starch 0.5%, glucose 2%, soya bean powder 1%, bacto peptone 0.2%, yeast extract 0.2%, NaCl 0.4%, K_2HPO_4 0.05%, $MgSO_4·7H_2O$ 0.05 %, and $CaCO_3$ 0.2%, pH 7.8
